# Fast long-term denudation rate of steep alpine headwalls inferred from cosmogenic ^36^Cl depth profiles

**DOI:** 10.1038/s41598-019-46969-0

**Published:** 2019-07-30

**Authors:** David Mair, Alessandro Lechmann, Serdar Yesilyurt, Dmitry Tikhomirov, Romain Delunel, Christof Vockenhuber, Naki Akçar, Fritz Schlunegger

**Affiliations:** 1 Institute of Geological Sciences, University of Bern, Bern, 3012 Switzerland; 2 Department of Geography, University of Zurich, Zurich, 8057 Switzerland; 3 Laboratory of Ion Beam Physics, ETH Zurich, Zurich, 8093 Switzerland

**Keywords:** Geochemistry, Geology, Geomorphology

## Abstract

Quantifications of *in-situ* denudation rates on vertical headwalls, averaged over millennia, have been thwarted because of inaccessibility. Here, we benefit from a tunnel crossing a large and vertical headwall in the European Alps (Eiger), where we measured concentrations of *in-situ* cosmogenic ^36^Cl along five depth profiles linking the tunnel with the headwall surface. Isotopic concentrations of ^36^Cl are low in surface samples, but high at depth relative to expectance for their position. The results of Monte-Carlo modelling attribute this pattern to inherited nuclides, young minimum exposure ages and to fast average denudation rates during the last exposure. These rates are consistently high across the Eiger and range from 45 ± 9 cm kyr^−1^ to 356 ± 137 cm kyr^−1^ (1σ) for the last centuries to millennia. These high rates together with the large inheritance point to a mechanism where denudation has been accomplished by frequent, cm-scale rock fall paired with chemical dissolution of limestone.

## Introduction

Denudation in its broader sense is a complex process, which is initiated and controlled by a variety of mechanisms and conditions such as chemical and physical erosion^1^, glacial sculpting^2,3^, tectonics^4^, and climate^5^. Our understanding of the spatial scale, rate and timing of denudation, is coined by a very large set of scale-specific data, quantified with a small number of methods. For mountain belts such as the European Alps, quantitative data on surface denudation is often inferred from concentrations of *in-situ* cosmogenic nuclides^6–9^, records of low temperature thermochronology^10,11^ and river sediment loads^12^. These data have also been used to measure the effects related to the coupling of individual erosional mechanisms^13–15^. Most of aforementioned studies share the same concept as they rely on sediment deposits of various type^7–9,16,17^ and scale as archives^6,18^. This inherently includes a possible bias because the original denudation signal may be blurred, or shredded, by transport mechanisms, intermediate storage, and mixing with material from other sources. Related possible pitfalls are already outlined in the pioneering studies where local headwall denudation has been quantified based on sediment budgets^16,19,20^. While low-temperature thermochronology techniques can yield information on local erosion rates of currently exposed bedrock, these methods are only suitable if the focus lies on erosion rates over millions of years. In the opposite cases, where remote sensing technologies were employed to measure surface changes at the local scale^21–23^, the timescales over which denudation was measured were too short to allow a meaningful interpretation of the underlying controls and mechanisms. Over the last decades, terrestrial cosmogenic nuclides (TCN)^24,25^ have been used to quantify the exposure history of rock surfaces, which built the basis for the temporal quantifications of coastal cliff collapse events^26^, rock fall activities in the high Alps^27^, and for the calculations of rock wall recession rates of limestone cliffs^28^. However, these studies were based on single TCN concentrations in surface samples only, which cannot be used to infer a time-averaged rate of denudation, as either a second TCN in the same sample or information on the TCN concentrations within depth profiles are needed for such an endeavor^25^.

**Figure 1:**
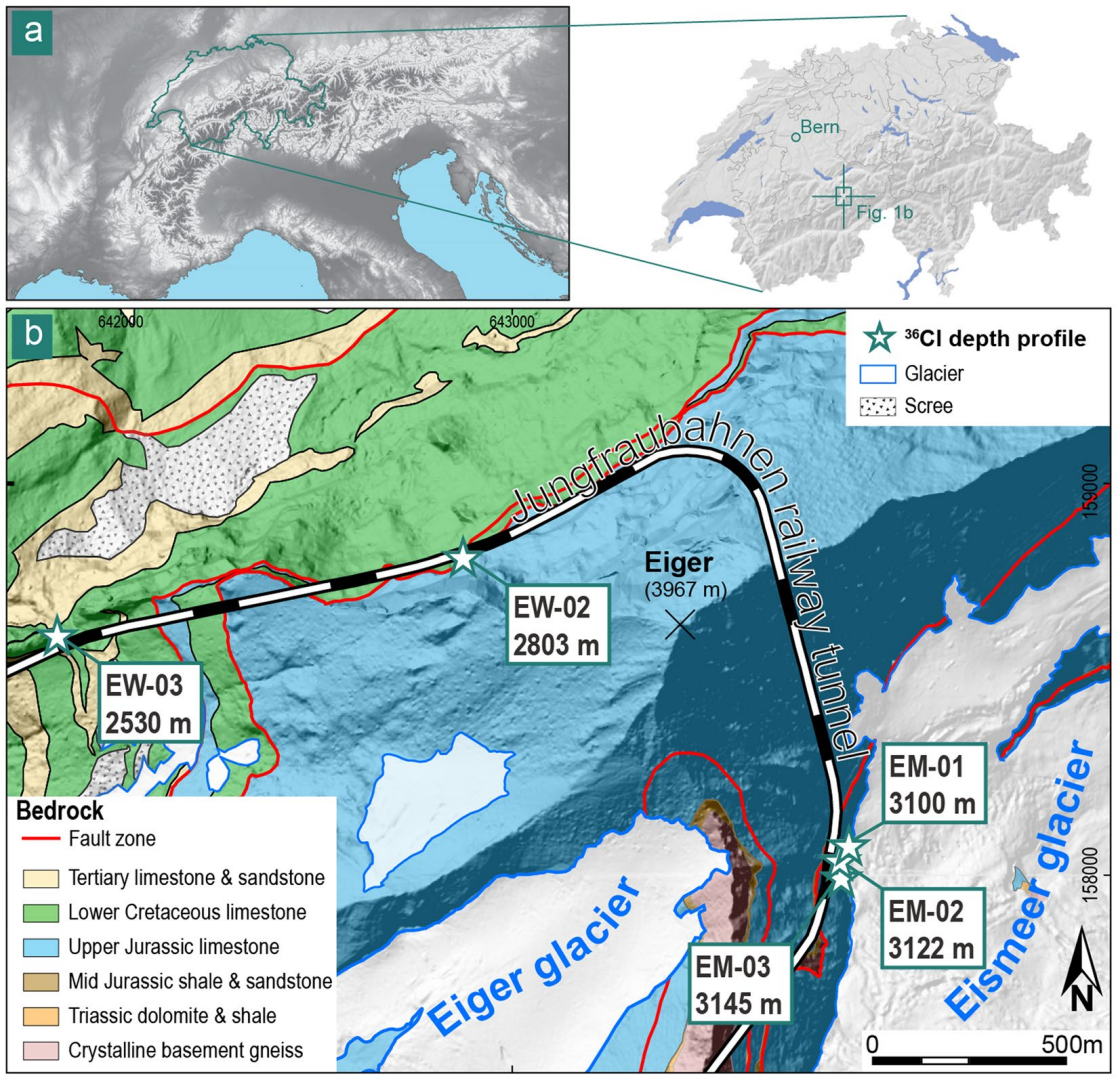
Geological setting of the study. (**a**) Regional map of the study area within the Central Alps of Switzerland (inserts). Basemap: hillshade based on the EU-DEM v1.1 with 25 m raster resolution, produced using Copernicus data and information funded by the European Union - EU-DEM layers. (**b**) Simplified geological map^33^, with depth profile sampling sites indicated. Basemap: hillshade based on the swissALTI3D DEM with 2 m raster resolution, reproduced by permission of swisstopo (BA 19051).

**Figure 2:**
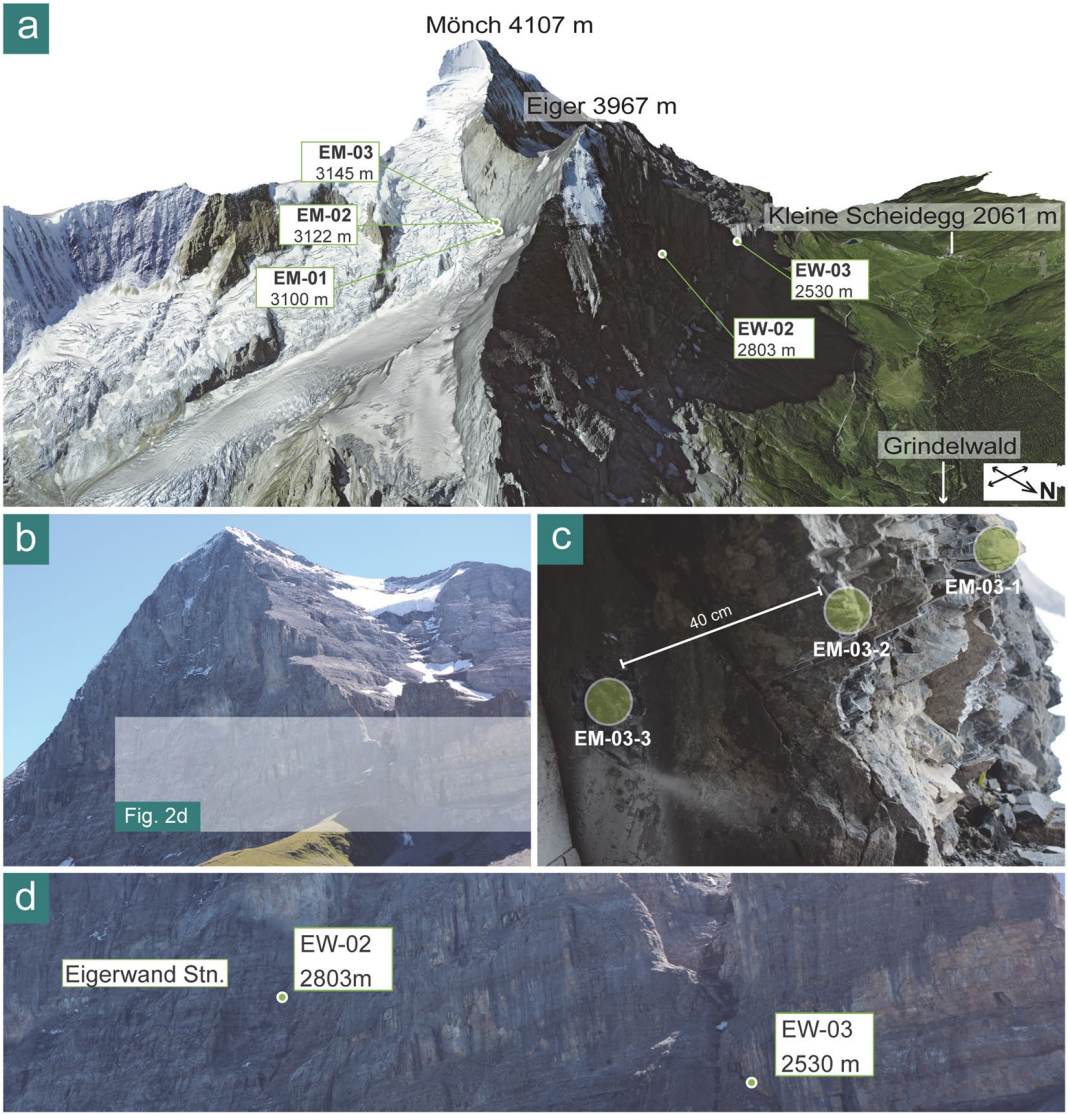
Geomorphic setting of the study area. 3D view (**a**) of the Eiger mountain (DEM and aerial image provided reproduced by permission of swisstopo; BA 19051) with NW face (**b**) and sample sites (**d**) within it. (**c**) Local bedrock surface formed by Jurassic limestone at sampling site EM-03.

**Figure 3:**
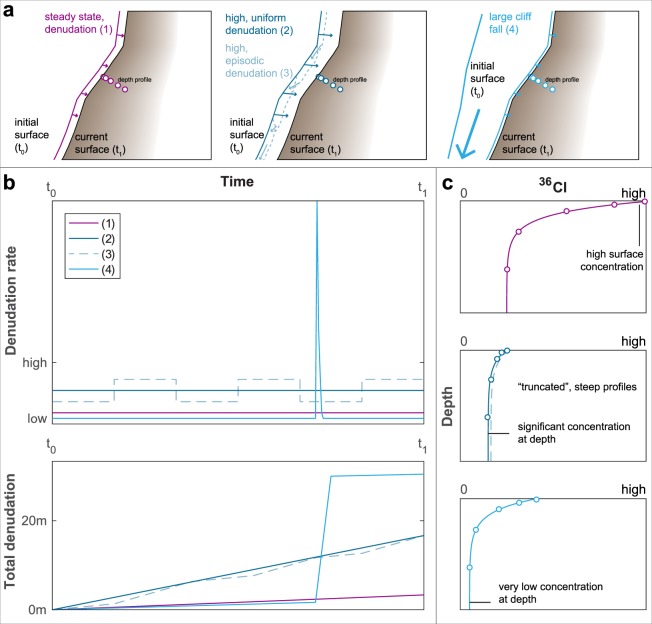
Schematic sketch of the effect of possible exposure scenarios (**a**) on the denudation rate and total cumulated net denudation over time (**b**) and on TCN profiles with depth (**c**) at the time t_1_. Key characteristics used to interpret our measured profiles are indicated and discussed in the text.

Here, we determine for the first time long-term denudation rates, including chemical erosion due to weathering, on a large vertical headwall by measuring and modelling concentrations of cosmogenic ^36^Cl along several depth profiles. We employ this methodology because it yields quantitative and *in-situ* information on the local denudation of the target headwall averaged over millenia^29–32^. We focus on the Eiger in the Central Swiss Alps (Fig. 1), a mountain where its NW face features one of the highest and almost vertical headwalls in the European Alps. This NW face is characterized by a 1800 m topographic relief, built-up by recrystallized fine-grained Jurassic limestone, recrystallized biogenic Cretaceous limestone and Tertiary shale, sandstone and limestone at its foot^33^. The mountain displays a pyramidal shape with several small cirque-glaciers on its SW and SE flank, and a narrow, steeply exposed ridge to the NE (Fig. 2). The NW face has an over-steepened and rough character compared to the comparatively smoother, yet equally steep southern flank, which hosts an active glacier at its foot (Fig. 1). We collected depth profile samples at five different sites within the NW and SE flank, where the elevations of the sample sites range from 2530 m a.s.l. in the NW face to 3145 m a.s.l. in the SE flank (Fig. 2; Table 1). We measured the ^36^Cl concentrations in the limestone bedrock samples with the Accelerated Mass Spectrometer (AMS) at the ETH Zürich. Principally, upon inferring denudation rates from TCN concentrations within depth profiles, one considers the time since the studied surface has been exposed to the cosmically induced particle shower, i.e., the exposure time, which started at an unknown point of time in the past (t_0_; Fig. 3). Potential TCN accumulation before that time is considered as an inherited contribution. For the case of bedrock headwalls, this could have only occurred through the removal of bedrock material, which was overlying the bedrock surface prior to t_0_. The investigated exposure time itself ranges from this starting point (t_0_) to the time of sampling (t_1_; Fig. 3). The long-term denudation rate therefore represents the averaged denudation of the bedrock integrated over this time period (Fig. 3). We obtained information on the concentrations of inherited ^36^Cl before the start of the current exposure (Fig. 3), minimum ages for the start of the current exposure history (t_0_) and denudation rates averaged between t_0_ and the present (time of sampling; t_1_) through Monte Carlo (MC) simulations of ^36^Cl concentration patterns within each depth profile^31,32^. By utilizing the shape of the TCN profiles within the bedrock, we can conceptually constrain the erosional mechanisms and history during this inferred exposure time (i.e, between t_0_ and the present; Fig. 3). This permits us, for the first time, to determine a rate at which denudation has operated on a vertical headwall over millennia.

**Table 1:** Site-specific parameters for the depth profiles. For a discussion of the profile geometry, see Supplements S1, S2.

Sample site	Long. [°]	Lat. [°]	Elev. [m]	S lope α [°]	Profile plunge β [°]	Dip dir. θ_0_ [°]	Shielding factor S_T_	Apparent att. length Λ_f_ [g cm^−2^]	Eff. app. att. length Λ_f,e_ [g cm^−2^]
EM-01	8.01075	46.57252	3100	74.8	5.0	96.4	0.6438	157.5	93.2
EM-02	8.01049	46.57180	3122	74.5	0.0	111.3	0.5754	157.5	99.0
EM-03	8.01043	46.57217	3145	80.9	0.0	99.4	0.5843	157.6	86.0
EW-02	7.99797	46.57922	2803	53.0	30.0	298.0	0.4888	156.8	145.0
EW-03	7.98443	46.57749	2530	83.3	3.5	7.0	0.4497	156.2	92.4

Shielding and attenuation lengths were calculated using the CRONUScalc v2.0 topographic shielding web calculator^59^.

## Results

### Concentrations of cosmogenic ^36^Cl

**Figure 4:**
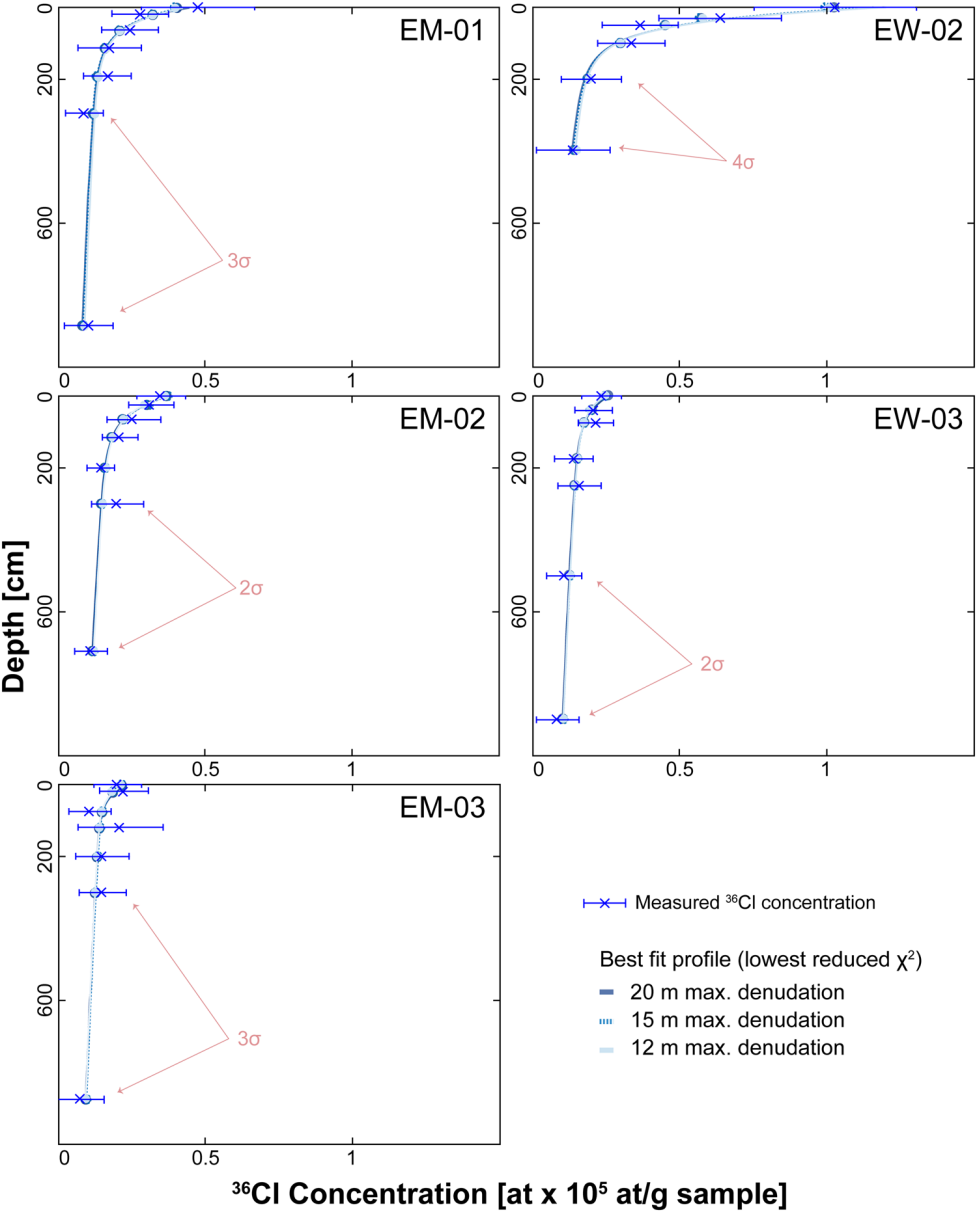
Measured ^36^Cl concentrations plotted against the best-fit profiles for each site and all corresponding modelling runs (superimposed). Measurement uncertainties indicated account for 2σ uncertainty for EM-01, EW-03, for 3σ for EM-01, EM-03 and 4σ for EW-02, respectively.

All 34 samples collected along five depth profiles have ^36^Cl concentrations that generally decrease with depth (Table 2). Only samples EM-03-01, i.e. the surface sample of the related profile on the southern side, and sample EM-03-03 (Figs 1, 2), feature lower concentrations than the subsequent deeper one (Fig. 4). One sample (EW-02-7) has a concentration with a large uncertainty (relative error at 1σ of 106%); this sample is thus excluded from the further analysis. The other 33 samples have ^36^Cl concentrations ranging between 0.07 × 10^5^ and 1.02 × 10^5^ at g^−1^ and relative uncertainties (1σ) between 7 and 39% (Table 2, Fig. 4). Samples from profiles EM-01, -02, -03 and EW-03 yield low ^36^Cl concentrations in near surface samples (≤ 0.47 × 10^5^ at g^−1^). The related ^36^Cl concentration patterns result in seemingly truncated profiles (Fig. 3), i.e., the near surface concentrations are lower than expected from concentrations at depth (Fig. 4). Only EW-02 displays higher near surface concentrations of 0.63 × 10^5^ to 1.02 × 10^5^ at g^−1^. Apart from this, we find no significant difference between the NW (EW profiles) and the SE sites (EM profiles). This includes the total ^36^Cl concentrations and the decrease in these values over depth. Samples at greater depth (>100 cm) show ^36^Cl concentrations between 0.07 × 10^5^ at g^−1^ and 0.21 × 10^5^ at g^−1^ (Table 2), which are high compared to near surface concentrations.

**Table 2:** AMS results for measured Cl isotope concentrations in all samples.

Sample	Dissolved sample [g]	depth [cm]	^35^Cl spike [mg]	^36^Cl/^35^Cl [10^−14^]	Total ^35^Cl [ppm]	^36^Cl [10^5^ g^−1^ sample]
EM-01-1	70.39	0	2.569	6.97 ± 0.85	6.37 ± 0.07	0.473 ± 0.061
EM-01-2	88.93	19.7	3.038	4.39 ± 0.41	6.06 ± 0.10	0.276 ± 0.029
EM-01-3	88.96	64.0	2.947	3.95 ± 0.41	5.30 ± 0.19	0.242 ± 0.029
EM-01-4	88.79	113.2	3.034	2.85 ± 0.47	5.52 ± 0.16	0.170 ± 0.033
EM-01-5	89.16	191.9	3.026	2.83 ± 0.32	6.06 ± 0.15	0.166 ± 0.023
EM-01-6	89.70	295.3	3.002	1.52 ± 0.22	6.31 ± 0.19	0.084 ± 0.018
EM-01-7	89.02	885.8	2.982	1.79 ± 0.33	5.39 ± 0.18	0.100 ± 0.024
EM-02-1	88.72	0	3.000	5.41 ± 0.53	7.07 ± 0.25	0.347 ± 0.037
EM-02-2	89.91	25	3.037	4.48 ± 0.43	12.34 ± 0.17	0.311 ± 0.033
EM-02-3	87.75	70	3.033	4.06 ± 0.61	5.76 ± 0.22	0.251 ± 0.042
EM-02-4	87.34	125	2.993	3.51 ± 0.35	4.57 ± 0.21	0.207 ± 0.025
EM-02-5	88.35	200	3.044	2.48 ± 0.23	4.33 ± 0.27	0.140 ± 0.018
EM-02-6	88.98	300	3.020	3.44 ± 0.61	3.95 ± 0.35	0.198 ± 0.040
EM-02-7	87.77	710	3.030	2.04 ± 0.33	3.27 ± 0.38	0.109 ± 0.023
EM-03-1	90.27	0	3.037	3.31 ± 0.32	5.68 ± 0.11	0.199 ± 0.023
EM-03-2	90.28	20	3.033	3.62 ± 0.34	5.99 ± 0.24	0.220 ± 0.025
EM-03-3	84.82	75	3.029	1.84 ± 0.26	3.86 ± 0.15	0.104 ± 0.020
EM-03-4	88.65	120	3.010	3.40 ± 0.66	6.01 ± 0.23	0.208 ± 0.045
EM-03-5	87.51	200	3.033	2.41 ± 0.37	6.61 ± 0.21	0.147 ± 0.027
EM-03-6	87.87	300	3.026	2.51 ± 0.31	4.79 ± 0.38	0.147 ± 0.023
EM-03-7	86.95	875	3.023	1.22 ± 0.27	11.38 ± 0.15	0.074 ± 0.023
EW-02-1	87.78	0	3.010	16.06 ± 1.01	4.63 ± 0.56	1.024 ± 0.066
EW-02-2	87.09	29.8	3.013	10.55 ± 0.79	1.96 ± 2.58	0.635 ± 0.050
EW-02-3	87.39	49.6	3.062	6.01 ± 0.44	2.50 ± 0.30	0.363 ± 0.030
EW-02-4	90.61	99.3	3.059	5.74 ± 0.39	2.39 ± 0.08	0.333 ± 0.026
EW-02-5	87.37	198.5	3.055	3.43 ± 0.32	1.83 ± 0.17	0.196 ± 0.023
EW-02-6	88.82	397.0	2.999	2.51 ± 0.45	1.74 ± 0.27	0.134 ± 0.029
EW-02-7	89.53	972.7	3.053	8.40 ± 8.63	3.16 ± 0.11	0.509 ± 0.540
EW-03-1	88.50	0	3.021	3.97 ± 0.44	2.92 ± 0.44	0.231 ± 0.029
EW-03-2	89.24	39.9	3.024	3.68 ± 0.41	1.35 ± 0.91	0.204 ± 0.027
EW-03-3	89.67	74.9	3.019	3.84 ± 0.40	1.42 ± 0.65	0.212 ± 0.026
EW-03-4	92.04	174.7	3.025	2.64 ± 0.45	1.48 ± 1.10	0.138 ± 0.028
EW-03-5	88.43	249.6	3.021	2.90 ± 0.50	0.78 ± 2.83	0.156 ± 0.032
EW-03-6	88.61	499.2	3.016	1.94 ± 0.35	3.28 ± 0.45	0.105 ± 0.025
EW-03-7	88.83	898.6	3.010	1.62 ± 0.49	1.19 ± 0.65	0.080 ± 0.031

Reported 1σ uncertainties account for AMS reproducibility and counting statistics on concentrations.

### Apparent surface exposure ages

Apparent exposure ages were calculated for surface samples by considering a simple end-member scenario consisting of one single exposure event, assuming no inherited ^36^Cl. This allows us to estimate minimum exposure ages of the rock surfaces at each of the profile sites. The resulting zero-denudation apparent minimum ages are younger than 2 ka (Table 3) and cluster between 0.17 ± 0.03 and 0.40 ± 0.07 ka, except for EW-02-1, which returns an age of 1.7 ± 0.3 ka. Apparent surface exposure ages, which were calculated under the assumption of steady state denudation at the rate of the local catchment^34^ (ε = 0.12 mm yr^−1^, inheritance = 0, single exposure) only increase the ages up to maximum of ~20%. An upper-end estimation on the exposure age can be constrained by calculating apparent exposure ages for the deepest available samples, thereby considering similar conditions (ε = 0.12 mm yr^−1^, inheritance = 0, single exposure). The resulting apparent ages range from 36 ± 18 to 103 ± 87 ka, overlapping within the 1σ confidence interval. These values represent upper boundaries for further MC modelling since they are based on the assumption that all ^36^Cl concentrations at deeper levels stem from one exposure, which would require a situation of a slowly eroding rock surface over one exposure period.

**Table 3:** Apparent exposure ages of the current surfaces from selected samples (surface samples and deepest samples of the profile, respectively) with 1σ total uncertainties, accounting for propagated uncertainties on all input parameters^59^ (Inh. = inheritance, ε = denudation rate).

Sample	App. Min. age (Inh. = 0, ε = 0) [ka]	App. Steady denudation age (Inh. = 0, ε = 0.12 mmyr^−1^) [ka]
EM-01-1	0.40 ± 0.07	0.42 ± 0.08
EM-02-1	0.29 ± 0.05	0.30 ± 0.05
EM-03-1	0.17 ± 0.03	0.18 ± 0.03
EW-02-1	1.73 ± 0.26	2.15 ± 0.42
EW-03-1	0.37 ± 0.07	0.40 ± 0.08
EM-01-7	40 ± 11	62 ± 26
EM-02-7	37 ± 9	60 ± 24
EM-03-7	29 ± 10	40 ± 18
EW-02-6	29 ± 7	54 ± 25
EW-03-7	55 ± 24	105 ± 87

### Profile modelling

Exposure age, denudation rate and inheritance can be estimated from randomized depth profile modelling^31^ with Monte Carlo (MC) simulations^32^. We modelled our ^36^Cl concentrations in the depth profiles by limiting the solution space as little as possible (see methods). This works well for estimations of denudation rate and inheritance, but not for the determination of mean or maximum exposure ages, which strongly depends on the assigned initial constraints on denudation^32^. In the absence of geological information, we assigned total maximum denudation cut-offs of 12, 15 and 20 m (see methods where the selection of these values is justified) for all profiles during independent model runs with the purpose to evaluate the dependency of our model outputs on the initial assumptions.

**Figure 5:**
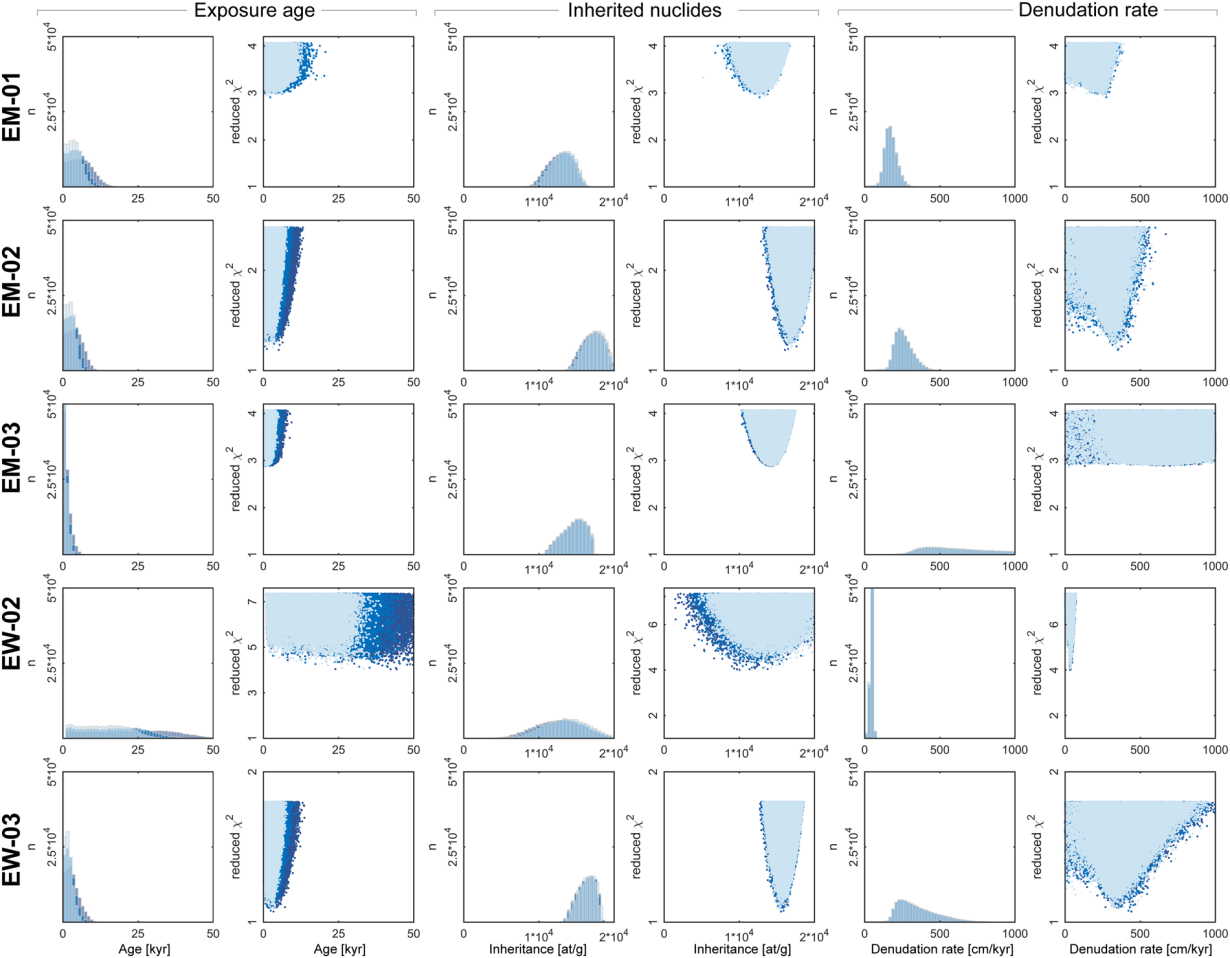
Exposure age, inheritance and denudation rate estimations for each profile from Monte Carlo (MC) depth profile modelling superimposed and color-coded for the different total max. denudation cut-offs (light blue = 12 m, blue = 15 m and dark blue = 20 m, respectively. For result summary see Table 4, for complete input, model setup and result including raw data used for the figure, we refer to Supplement S6.

All 10^5^ modeled ^36^Cl concentration depth profiles for each site for EM-02 and EW-03 are within the 2σ confidence interval of the measured ^36^Cl concentrations, while EM-01 and EM-03 are within 3σ, and EW-02 is within 4σ only (Fig. 4). The corresponding reduced minimum chi-square (χ^2^) values show a similar trend, where the lowest values are close to one for EM-02 (1.2) and EW-03 (1.1) and are slightly higher for EM-01 (2.9), EM-03 (2.9) and EW-02 (4.0). We note here, that despite needing a larger solution space for reaching 10^5^ good fits between simulation results and data, the best fitting model profiles for EW-02 fall within the 3σ confidence interval of the measured ^36^Cl concentrations. The results for exposure age, inheritance and denudation have similar χ^2^ distributions with clear minima for all model runs (Fig. 5). Looking closer, the denudation rate and inheritance estimates that result from these simulations do not significantly change with variation of the possible maximum estimates of cumulative denudation (cut-off values of 12, 15 and 20 m; see Fig. 5 and methods). Most best-fit estimates (i.e., featuring the lowest χ^2^ value; Table 4) for denudation rate and inheritance fall close to or within the uncertainty range of the mean. Hence, we find reasonably well-defined Gaussian probability distributions for inheritance and denudation rate for profiles EM-01, EM-02, EW-02 and EW-03 (Fig. 5). However, profile EM-03 results in a strongly skewed distribution of the modeled parameters (Fig. 5). Aside from that, we find that profiles EM-01, EM-02, EM-03 and EW-03 yield resembling denudation rate and inheritance distributions, while the pattern of EW-02 differs. We proceed by first presenting the results of the four similar profiles, before discussing the results of EW-02 separately.

**Table 4:** Result statistics for the different Monte Carlo (MC) model runs.

Profile		Age	ε	Inh.	Age	ε	Inh.	Age	ε	Inh.
		ka	cm/kyr	at/g	ka	cm/kyr	at/g	ka	cm/kyr	at/g
		12 m max. denudation			15 m max. denudation			20m max. denudation		
EM01	MEAN	3.7	172.0	13184	4.6	173.3	13022	6.1	174.7	12826
	STD	2.2	42.9	1483	2.7	42.3	1525	3.7	41.7	1560
	MEDIAN	3.6	169.1	13273	4.4	170.1	13107	5.8	171.2	12911
	Min	0.1	0.0	5192	0.1	0.0	5192	0.1	0.0	6818
	lowest χ^2^	1.6	254.0	13326	2.3	275.2	12712	11.7	164.2	11318
	Max	19.4	390.4	16686	20.6	383.4	16680	25.3	409.6	16641
EM02	MEAN	2.5	257.6	17351	3.1	259.7	17261	4.1	262.0	17155
	STD	1.5	65.5	1300	1.8	64.3	1324	2.4	63.8	1350
	MEDIAN	2.4	250.9	17413	2.9	252.4	17324	3.9	254.0	17222
	Min	0.1	0.1	13191	0.1	0.2	13237	0.1	0.2	12911
	lowest χ^2^	3.1	341.2	16913	1.2	355.0	16489	5.1	352.4	16068
	Max	8.4	583.8	20539	10.0	602.1	20539	13.2	670.7	20509
EM03	MEAN	0.8	935.8	14689	1.1	943.8	14649	1.4	948.5	14609
	STD	0.7	549.2	1515	0.9	550.5	1530	1.2	547.6	1541
	MEDIAN	0.6	772.4	14850	0.8	778.5	14812	1.1	784.4	14779
	Min	0.0	0.2	10274	0.0	1.5	10274	0.0	0.2	10344
	lowest χ^2^	0.1	23.9	14608	0.1	23.9	14608	0.1	132.1	14462
	Max	5.1	2499.9	17544	7.2	2499.9	17544	9.4	2499.9	17491
EW02	MEAN	13.6	45.4	13625	17.0	45.8	13184	22.8	46.1	12610
	STD	7.7	8.7	2933	9.6	8.3	3040	12.9	7.9	3152
	MEDIAN	13.4	46.1	13669	16.9	46.3	13234	22.7	46.4	12656
	Min	1.0	0.1	2715	1.0	0.2	2715	0.9	0.1	72
	lowest χ^2^	33.4	34.6	13295	35.3	37.5	11664	54.0	33.3	10042
	Max	45.3	73.3	23137	50.2	73.3	23137	69.0	71.3	23428
EW03	MEAN	2.0	350.1	16353	2.5	352.6	16304	3.3	355.9	16254
	STD	1.3	135.3	1107	1.6	135.5	1127	2.2	136.6	1149
	MEDIAN	1.8	321.8	16444	2.2	324.0	16398	2.9	326.1	16357
	Min	0.1	0.2	12955	0.1	0.1	12740	0.05	0.1	12772
	lowest χ^2^	2.6	347.5	15424	2.5	422.3	15894	3.4	441.9	15549
	Max	8.0	1106.7	18535	11.3	1110.4	18555	13.7	1105.5	18533

Complete MC inputs, setups and results are given in Supplement S6 (Inh. = inheritance, ε = denudation rate).

The profiles EM-01, EM-02 and EW-03 yield minimum ages for the 12 m total net denudation model run of 0.1 ka. The minimum ages remain constant for all three maximum denudation cut-off setups (Table 4). Median ages range between 1.8 and 3.6 ka for the 12 m cut-off model run. The median ages then substantially increase with larger estimates of cumulative denudation, leading to a range between 2.2 and 5.8 ka for 20 m of net denudation (Table 4). The distributions of the modelled ages thus change depending on the applied denudation cut-off (Fig. 5, Supplement Fig. S6). Regarding the model outcome for the denudation parameter, the same three profiles return consistent mean values ranging from 172 ± 43 to 350 ± 135 cm kyr^−1^ for the 12 m net cut-off setup, while EM-03 shows a higher value and a larger relative uncertainty (936 ± 549 cm kyr^−1^). The modelled denudation rate probability distributions are consistent for all three estimates of cumulative denudation (Fig. 5), in the sense that they closely agree within uncertainty with each other, and therefore are independent of the applied denudation cut-off. Inheritance of ^36^Cl is significant for the 12 m net denudation model (mean ranging from 1.3 × 10^4^ to 1.7 × 10^4^ at g^−1^) with minima of 5.2 × 10^3^ to 1.3 × 10^4^ at g^−1^. Maximum modelled inheritance ranges from 1.7 × 10^4^ to 2.1 × 10^4^ at g^−1^, which would account for a significant proportion of the concentrations that were measured for the corresponding surface sample (e.g. > 60% for EW-03-1). The inheritance distributions and their mean values are independent of the total net denudation, as the corresponding values remain constant with a larger total net denudation cut-off (Table 4).

The modeling results of EW-02 show the same systematic trends as the other depth profiles, yet this profile returns significantly different results. Minimum ages are close to 1 ka for all three cumulative denudation cut-offs. Again, the median of the modelled ages changes with these cut-offs, ranging from 13.4 to 22.7 ka. Median denudation rates range from 46.1 to 46.4 cm kyr^−1^ for all cut-off setups. Estimates for the mean inheritance cluster around 1.3 × 10^4^ at g^−1^. In addition, for EW-02, inheritance and denudation rate ranges and distributions are independent of the applied values of cumulative denudation (Fig. 5).

## Discussion

Young apparent surface exposure ages of surface samples (Table 3) in combination with relatively high nuclide concentrations at depth (Table 2) provide a challenge for constraining the denudation rates on rock walls when TCN depth profiles are employed. However, they also provide a unique opportunity to infer information on the long-term average denudation rate, after considering three method-specific issues. We will first address these three points, thereby justifying the validity of our selected approach upon designing the sampling strategy. We then proceed to discussing the implications arising from the concentration patterns in the depth profiles and from the Monte Carlo (MC) modelling thereof, before we outline the results in a broader context regarding the rates and the mechanisms of headwall denudation.

First, the selection of the sampling sites was constrained by the cavities that link the railway tunnel with the surface of the Eiger, leading to unconventional depth profile geometries (not vertical and not precisely centered below one point). Nevertheless, by adapting the shielding^25,35,36^ correction at the Eiger such as that the correct nuclide production through spallation is considered, and by considering the small vertical offsets under a uniform surface assumption^35,37^, these effects can be considered as negligible (Supplements S1, S2). Second, since the tunnels were constructed between 1896 and 1905 AD through conventional tunneling with handheld drills and explosives, the original bedrock texture near the exits could have been altered or even destroyed. We cannot completely exclude this possibility, but we sampled only intact bedrock with no signs of artificial destruction. Furthermore, we can confidently exclude the occurrence of artificial erosion for at least 4 of the 5 profiles because: (i) samples at depth yield high concentrations, which most likely points to inherited ^36^Cl nuclides (see below); and (ii) apparent exposure ages are significantly older than 0.11 ka for all profiles, also verified by the MC profile modelling (Table 4). The only profile that can be modeled with minimum ages less than 100 years is EM-03. Furthermore, because of the ^36^Cl concentration being low on the surface in comparison to samples at greater depth, and since the modeled range for the denudation rate estimate is quite large, we do not consider profile EM-03 to represent a fully intact bedrock surface. Therefore, we base our interpretation on the results of the other four profiles EM-1, EM-2, EW-02 and EW-03. Third, another bias could be introduced by the seasonal snow cover, as this has a significant effect on the ^36^Cl production by thermal and epithermal neutrons, that are captured by ^35^Cl^38,39^. Therefore, a correction factor related to a seasonal snow cover is commonly considered^37^. We refrained from such a correction because we sampled steep rock walls (slope >50°; Table 1) where snow is unlikely to accumulate over extended periods, and our samples show very low concentrations of natural ^35^Cl (Table 2).

We now proceed discussing the implications arising from the concentration patterns in the depth profiles. In particular, at all sites, the measured TCN concentrations are consistently decreasing with depth (Fig. 4). We also find high ^36^Cl concentrations at depths exceeding 3 m (Table 2), which are not in agreement with the young apparent surface exposure ages for a simple exposure scenario with zero or a low steady state denudation rate (Table 3; Fig. 3). Thus, a significant part of these nuclides has built up at depth throughout a long-term history of exposure, i.e. before t_0_ (Fig. 3). This implies that our apparent exposure ages, which have been derived from surface samples, are most likely only close to a minimum age due to a more complex exposure history. In the next section, we address this point and we particularly use the ^36^Cl concentrations in the depth profiles for extracting information on the inherited ^36^Cl and denudation rate through modeling.

MC modelling results of TCN concentrations in depth profiles have been used in recent years to estimate these parameters in question^32^ by using site specific geological constraints. We do not have precise constraints, but we can use the specified confidence interval and the resulting χ^2^ cut-off values to test the validity of the results that came from the depth profile modelling. These show that at all sites, enough model profiles (10^5^) could be obtained for a meaningful parameter estimation within a reasonable sigma range^32^. For 4 out of 5 sites, the model profiles displaying the lowest χ^2^ values could reproduce the depth profile data within or close to the 2σ range (Table 4). Furthermore, MC modelling of TCN concentrations works well for depth profiles where the bedrock has a homogeneous chemistry and where the exposure history of the corresponding surface has been fairly simple without the occurrence of partial burial^29,32^. Concerning the chemical homogeneity, all profiles feature a rather pure limestone composition except for profile EW-02 (Supplementary Table S3), which shows some variation in quartz content. Compared to the other four profiles, this is reflected by the larger χ^2^ solution space needed to obtain 10^5^ accepted fits between model runs and data. With respect to the exposure history, we can exclude burial in the past because the over-steepened bedrock surfaces prevent the accumulation of material. Accordingly, the only mechanism exposing fresh rock at the surface includes the removal of the previous surface through mass wasting processes of various magnitudes and scales (Fig. 3). Finally, and probably most important, although we have no specific a priori and precise information on the exposure age, the denudation rate and the inheritance (see above), we can safely employ, as boundary conditions, the here proposed cumulative denudation values that we derived from ^36^Cl production systematics without distorting the results (Supplement Fig. S6). In particular, by selecting maximum net denudation cut-offs of 12, 15 and 20 m as constraints, we cover a large range of realistic denudation scenarios, conditioned and thus controlled by nuclide production systematics (see method section for justification). Additionally, we can only reliably infer minimum exposure ages from the MC modelling^32^ of such a dataset. However, the minimum ages agree well with both sets (zero erosion and steady state denudation) of apparent exposure ages from the same surface samples (Table 3). In addition to the aforementioned model constraints, we can use geological information to infer uppermost limits to a possible exposure age to test our results. These limits are 12 ka (Younger Dryas glacial advance^40^) in the south and to 19 ka (LGM deglaciation^41^) in the north face, at least for the last exposure. This is based on the assumption that during these periods the reconstructed ice cover was thick and erosive enough to reset the TCN clock.

**Figure 6:**
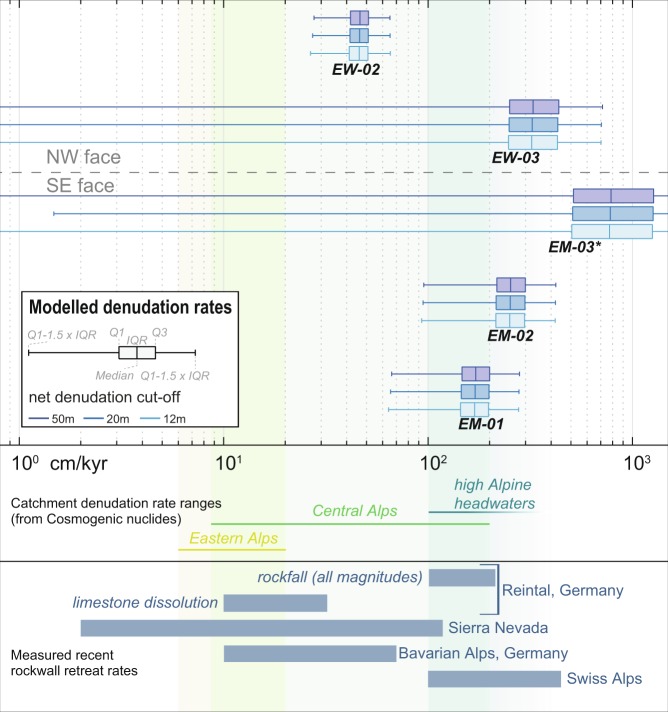
Modelled denudation rates from ^36^Cl depth profiles compared to catchment wide denudation rates for the Eastern Alps^71^, the Central Alps^6,9,17^ and high Alpine headwaters^7,8^, as well as selected alpine rock wall recession rates^16,42,72^. *EM-03 is suspected to be affected by human construction work and therefore not considered in our interpretation (see discussion in the text).

The MC modelling results have two major implications regarding our understanding of the rates and mechanisms of erosion operating at the Eiger, one of the steepest headwalls in the European Alps. First, the resulting models indicate the occurrence of significant inheritance at all sites (Table 4, Fig. 5) and for all model runs. This confirms the interpretation of high nuclide concentrations at deeper levels (Table 2) as inherited. Hence, we can first rule out a large rock fall event (≫ 20 m rock thickness removed at once) as a starting process before t_0_. Such a scenario would imply that our samples had been located at depths, were production is too low to produce the excess of nuclides we have measured in our deepest samples (Fig. 3). This interpretation is also supported by the general absence of rock fall deposits at the foot of the walls^33^. Second, the modelled denudation rates, ranging from 172 ± 43 to 356 ± 137 cm kyr^−1^, for profiles EM-01, EM-02 and EW-03. (Fig. 6), show a remarkable consistency on both flanks, thus pointing to a scenario where the entire Eiger has experienced fast denudation for at least the past 200 to 1000 years. These findings align particularly well with recent findings of high retreat rates in cliffs^20,42^, further they align with recession rates for steep rock walls^16^, and steep alpine catchments close to headwalls^8^. However, they fall in the upper range of the values reported for the scale of individual catchments (up to 150 ± 50 cm kyr^−1^)^7^ and for glacial/periglacial environments in the Central Alps where average denudation rates range between 1 and 2 mmyr^−1^ (100–200 cm kyr^−1^)^6,43^. An exception is site EW-02, situated at a higher elevation within the NW face, where modeling implies a lower mean denudation rate between 45 ± 9 and 46 ± 8 cm kyr^−1^ for 1 kyr, or possibly even longer. This profile is the only one, where the modelled average denudation rate could correspond to the rate of carbonate dissolution and frost weathering alone^42,43^ (Fig. 6). The other three profiles record values that are much higher, which might hint towards a scenario of an enhanced footwall erosion. Such high rates are impossible to reflect solely the occurrence of chemical weathering and erosion^44^, frost weathering by ice segregation^1^ and subsequent erosion or even glacial abrasion^43^. Although chemical and physical weathering might play a substantial role^42^ in the preconditioning slopes for failure, the effective process for such settings is most likely rock fall of various magnitudes. Because we can exclude the occurrence of large-scale, high magnitude bergsturz and cliff fall^42^ events to explain the cosmogenic records, a scenario where surface erosion has been accomplished by high-frequency, small-magnitude rock fall processes^20,45^ offers the best explanation for maintaining high denudation rates and ultimately producing the measured nuclide inventory.

In summary, the presented *in-situ* bedrock denudation rates on the Eiger are among the highest that have been measured so far in an Alpine environment. The high concentrations of inherited cosmogenic ^36^Cl excludes the possibility that these fast rates have been accomplished through large-scale mass wasting processes such as cliff falls. We rather consider a crumbling mechanism where cm-to dm-scale bedrock chips are removed from the bedrock surface at high frequency, which in combination with limestone dissolution might accomplish the headwall retreat of the Eiger from both sides at very high rates.

## Methods

Cosmogenic ^36^Cl is produced from cosmic rays by (1) spallation mainly from Ca, K, and less through Fe, Ti, (2) low energy (thermal and epithermal) neutron absorption by ^35^Cl, and by (3) fast muon interaction and slow muon capture^25^. This production is thus dependent on the chemical composition of the individual samples^25,46^. In limestone, spallation from Ca is the predominant production reaction^47,48^, while muogenic production from Ca becomes important with increasing depth^49–51^. The *in-situ* production is dependent on the sample position, which thus needs a scaling to geographic position, elevation, and correction for shielding from cosmic rays^25,37^. The measured concentrations of ^36^Cl and the robustness of the subsequent interpretations thus hinge on site and sample specific conditions, and it relies on the selection of the corresponding production calculation method.

### Sampling and measurements of ^36^Cl concentrations

Seven bedrock samples, which includes 1 surface sample at each site, were collected along 5 depth profiles with a battery-powered saw, hammer and chisel following standard sampling guidelines^52^. Each sample was about 5 or 10 cm thick and consisted of 1 to 1.5 kg of bedrock. Depth profile sampling was possible through the occurrence of sub-horizontal, several tens of meter short tunnels that link the railway tunnel with the surface of the bedrock. These tunnels were originally used to dispose rock material during the construction of the railway tunnel and were constructed along the shortest path to the surface. We sampled material along the lateral walls of the several meter-high and -wide tunnels (see Supplement Fig. S1 and Supplement Table S2). All parameters characterizing the sampling site are presented in Table 1; detailed information about profile geometries and shielding is given in Supplement S1.

Sample preparation for *in-situ* ^36^Cl whole-rock analyses followed state-of-the-art routines^53,54^, which base on the method of Stone (1996)^47^. This includes whole rock crushing and sieving to recover the 250–400 mm grain size fraction, two steps of HNO_3_ leaching and rinsing with ultra-pure water, and addition of a ^35^Cl carrier. The samples were then dissolved with HNO_3_, precipitated with AgNO_3_, and filtered by centrifugation. BaSO_4_ precipitation was done in order to remove ^36^S. The complete sample preparation took place at the Institute of Geological Sciences, University of Bern.

^35^Cl and ^36^Cl concentrations were measured at the ETH AMS facility of the Laboratory for Ion Beam Physics (LIP) with the 6 MV TANDEM accelerator, using an isotope dilution method^55,56^. ^35^Cl is assumed to represent total Cl content, which should be determined on unprocessed bulk material. This is justified by the overall low Cl content in the samples and their mineralogical homogeneity. Measured sample ratios were normalized to internal standard K382/4 N with a ^36^Cl/Cl ratio of (17.36 ± 0.36) × 10^−12^, and a stable ratio for ^37^Cl/^35^Cl of 31.98%^57^. Full process chemistry ^36^Cl/^35^Cl blank ratios of (2.9 ± 1.8) × 10^−15^ were used for correction, amounting to an adjustment of <15% for most samples, with a maximum value of 23.5% (EM-03-7). Major and trace element concentrations, which are required to calculate ^36^Cl production, were determined on separated 12 g aliquots of etched sample material at the ACTIVATION LABORATORIES LTD (Canada) using an inductively coupled plasma mass spectrometer (ICP-MS). These measurements were conducted on lithium metaborate/tetraborate fused samples (FUS-ICP-MS) for major oxides and trace elements respectively (chemical data are given in Supplementary Table S3). Sample EM-01-1 was separately measured, where sodium peroxide oxidation (Na_2_O_2_) instead of lithium metaborate/tetraborate was used as flux. Boron levels were measured by Prompt Gamma Neutron Activation Analysis (PGNAA). Uncertainties on reported concentrations (Table 2) account for AMS reproducibility, counting statistics and standard 1σ - error on concentrations.

### Surface exposure age calculation

The geometries of the depth profiles required the consideration of shielding effects^25,35–37^, which includes a topographic component, such as large obstacles (e.g. neighboring peaks), and a geometrical component arising from the slope of the sampled surface itself. A total shielding factor (*S*_*T*_) was calculated through the scaling of the nuclide production rate to the specific sampling site at the surface^25,35^. This was done through the consideration of an open sky visibility (as zenith angle) using the ‘skyline graph’ standard routine of ESRIs ArcGIS^TM^ Desktop 10.1 licensed to the Institute for Geological Sciences, University of Bern. The calculation was done for 1° azimuthal increments using a high-resolution DEM (2 m resolution) provided by the Swiss Federal Office of Topography, Swisstopo. We corrected the attenuated production from spallation^25,58^ at depth below surface using a site-specific effective apparent attenuation length (Λ_*f,e*_). Both parameters were calculated with the CRONUS Earth online Topographic Shielding Calculator v2.0 (http://cronus.cosmogenicnuclides.rocks/2.0/html/topo)^59^ based on previous versions^25,37^. Apparent attenuation lengths are calculated therein using the analytical PARMA model^60^ for the cosmic ray spectra in the atmosphere^25^. For a discussion and evaluation of our shielding correction approach, see Supplement S2.

Apparent surface exposure ages were calculated with the CRONUScalc web calculator v2.0^59^ resulting from the CRONUS earth project^61^. Results were obtained using production pararameters from previous work^47^ that have been re-evaluated^62^. This includes a SLHL spallogenic production rate of 52.2 ± 5.2 at ^36^Cl (g Ca)^−1^ yr^−1^, and 150 ± 15 at ^36^Cl (g K)^−1^ yr^−1^, and a low energy production rate of 696 ± 185 neutrons (g air)^−1^ yr^−1^. The production rate scaling followed Stone^63^, which itself is based on the method of Lal^24^, and considers a scaling of the ^36^Cl production according to the sample’s longitude, latitude and atmospheric depth. The production rates and the scaling are in good agreement^64^ with the recently published nuclide-specific scaling^38^. Apparent exposure ages (single exposure, zero inheritance, denudation rate ε = 0) were calculated for each sample considering the samples’ chemical composition (see Supplement Table S2). Rock bulk density was set to 2.68 ± 0.02 g cm^−3^ based on bulk density measurements (Supplement S4) of the same rock types^33^. Porewater contents could not be determined. We thus employed a conservative estimate of 2.3%, thereby assuming that the pores of the measured porosity (Supplement S4) were completely saturated. However, water content mainly affects ^36^Cl production through thermal and epithermal neutron capture on ^35^Cl near the surface^65^. As a consequence production by thermal and epithermal neutrons does not contribute significantly to the production of ^36^Cl in our case^46^ because of the low level of natural chlorine (typically <10 ppm; Table 2) and low levels of potassium (see Supplement S3). Minimum exposure ages were first calculated assuming a simple scenario (single exposure, denudation rate ε = 0, inheritance = 0). The exposure ages under the assumption of single exposure with a denudation rate of 0.12 mm yr^−1^ (derived from the local scale catchment^34^) and zero inheritance (Table 3) were calculated to test if a simple scenario with a steady state constant denudation rate could reproduce our measured ^36^Cl concentration within the depth profiles.

### Depth profile modelling

Exposure ages, surface denudation rates and inheritance were modelled from nuclide concentrations at depth using a Monte Carlo (MC) randomization approach^31^ trough a modified PTC^TM^ Mathcad^TM^ code^32^. This code was updated with production equations for ^36^Cl^46,66,67^ for neutrons and muons^50,51^ (for a muon fit to a depth of 30 m), in close agreement with production rate schematics reported for the CRONUScalc program^57^. For consistency, we used the same production rates as for the surface exposure age calculation^47,62^ (see also above). We also updated the shielding macro^36^, and we scaled the ^36^Cl production^24,63^ accordingly. We refrained from a global muon attenuation length fitting of the profile data^59,68^, instead we calculated the corresponding patterns for each modelled profile, for which we used a muon propagation parametrization based on experimentally determined muon stopping power^50,51^. This parametrization originally included empirically fitted parameters that led to significant overestimations of ^36^Cl production by muons for geological settings, especially at depth^68,69^. To avoid this bias, we adapted the approach of the CRONUS-Earth project^59,61^. We thus employed the parameters derived from this project, which are based on calibration profiles. For ^36^Cl, the corresponding values are *α* = 1 for the energy-dependent coefficient of the muon energy cross section^59,69^ and *σ*_0_ = 8.3 × 10^−30^ cm^2^ for the nuclide production cross-section in 10^−24^ cm^2^ at 1 GeV through fast muons^62^. We used the same value for the target elements Ca and K due to the good agreement within errors for the reported values^62^. For slow muon production, we again used a rate of 696 ± 185 neutrons (g air)^−1^ yr^−1^for the epithermal neutron production rate at the rock/air interface. Finally, effective probability values for particle emission to the nuclide of interest after capture of ${f}_{Ca}^{\ast }$ = 0.014 and ${f}_{K}^{\ast }$ = 0.058 for Ca and K, respectively^62^ were used. We additionally used the calculated shielding factor (*S*_*T*_) for corrections on spallogenic and muogenic production and apparent attenuation length for spallogenic production (Λ_*f,e*_) as input (see Supplement S2 for discussion). Corrected muon fluxes were subsequently used to calculate muon-induced neutrons. We further employed again a uniform water content (2.3%) and rock density (2.68 ± 0.02 g cm^−3^). Each profile was parametrized using a profile specific density and porosity value (“soil” in the input), whereas for the chemical composition an unweighted average of all samples of the same profile was employed. This is justified by the homogenous chemical composition of the bedrock at all sites (Supplement S3). Only section EW-02 features a change in SiO_2_ and CaO content below EW-02-3 (i.e. the uppermost samples are enriched in quartz compared to limestone).

For the modelled unknowns, computational constraints were put on: (1) exposure age, thereby using the apparent exposure age of the deepest sample as a maximum constraint for an exposure age; (2) maximum denudation rate during the exposure time interval, by leaving the denudation rate virtually unconstrained (i.e. an uppermost limit was set to 1500 or 2500 cm kyr^−1^) and by assuming a maximum thickness of eroded bedrock of 12, 15 and 20 m since the starting time t_0_; (3) inheritance, by using the ^36^Cl concentration of the surface sample as a largest possible value and zero as lower bound. The inferred maximum thickness of eroded bedrock of 12, 15 and 20 m since the starting time t_0_ were derived based on the following three considerations. (i) At all sites, the deepest samples were taken at depths close to or exceeding 7 m and consequently, the production of ^36^Cl almost exclusively has occurred by muon pathways. (ii) The muogenic production at these depths is in the order of 0.5 to 2% of the total surface production^50,68^, assuming a simple exponential muon attenuation length (Λ_*μ*_). (iii) Considering such an attenuation, that scales exponentially^24,25^ with reported muon attenuation lengths (Λ_*μ*_) between ~4000 and 5300 ± 950 g cm^−2^ for 2.7 g cm^−3^ rock density^50,68^, this translates to depths of ~15 and ~19 m for 1 Λ_*μ*_ and ~30 to ~38 m for 2 Λ_*μ*_, and accounts for a reduction of muogenic production by ~63% and ~87%, respectively. This means that independent of the attenuation length, our deepest samples would have to be located at a depth of >27 m at the time of t_0_ to allow for more than 20 m of total erosion. Any potential nuclides inherited from before t_0_ would then have accumulated at this depth or even deeper at a rate of <13% of the muogenic surface production.

We acknowledge that the consideration of a fixed attenuation length is a too simplistic approach upon estimating the nuclide production at depth with MC simulations^37,70^. Therefore, we iteratively adapted the attenuation length during the MC. We used a fixed attenuation length only to estimate realistic maximum cumulative denudation values, referred to as cut-off values, during the time interval between t_0_ and t_1_. Accordingly, by choosing 12, 15 or 20 m as cut-off values, we can test the robustness of our model and show the insensitivity of our inferred denudation rates on the depth range constraints. Following this consideration, possible inherited ^36^Cl concentrations were modelled for the surface sample (C_*inh*_) for each MC run, while the inheritance at depth (C_*inh,z*_) was parametrized following

$${C}_{inh,z}={C}_{inh}\cdot {e}^{(-\frac{Z}{{{\rm{\Lambda }}}_{inh}})},$$

where Λ_*inh*_ scales the inherited concentration at depth, based on a site-specific average length for muon attenuation^68,69^ (Supplement S6). This is justified since any ^36^Cl concentrations inherited from before t_0_ had to be accumulated at depths, where only production by muons is possible.

The general model acceptance was ideally confined to a 2 *σ* interval of measurement uncertainty, and it was increased first to 3 *σ*, and then 4 *σ* if not enough (10^5^) solutions were found within 2 *σ*. We note here that for EW-03 a cut-off of χ^2^ = 1.8 (corresponding to ~1.5 σ interval) was employed to sample only the solution space of the best fitting profiles. We report the resulting χ^2^ minimum values in Table 4 (all input parameters in Supplement S6). MC simulations were stopped after reaching 10^5^ profiles within the desired confidence (i.e., a χ^2^ lower than the defined cut-off). We present the total number of simulated depth profiles along with all model input and results in Supplement S6.

## Supplementary information

Supplementary Notes

Supplementary Dataset

## Data Availability

All data not directly reported in the manuscript are provided in the Supplement (i.e., chemistry, density, apparent exposure ages, and MC raw results).

## Code Availability

The code used for MC modelling, which is a modified version of the Mathcad^TM^ file from Hidy *et al*.^32^ is provided in the Supplement.
