# Supplementary material for: Fast long-term denudation rate of steep alpine headwalls inferred from cosmogenic 36Cl depth profiles
Source: Sci Rep. 2019 Jul 30;9:11023. doi: 10.1038/s41598-019-46969-0 (PMC6667707; doi:10.1038/s41598-019-46969-0)
Supplement: Supplementary file 1 — Supplementary Notes [file 41598_2019_46969_MOESM1_ESM.docx]

Supplement for: Fast long-term denudation rate of steep alpine headwalls inferred from cosmogenic ^36^Cl depth profiles

David Mair^1^*, Alessandro Lechmann^1^, Serdar Yesilyurt^1^, Dmitry Tikhomirov^1,2^, Romain Delunel^1^, Christof Vockenhuber^3^, Naki Akçar^1^, and Fritz Schlunegger^1^

^1^Institute of Geological Sciences, University of Bern, Bern, 3012, Switzerland

^2^Department of Geography, University of Zurich, Zurich, 8057, Switzerland

^3^Laboratory of Ion Beam Physics, ETH Zurich, Zurich, 8093, Switzerland

# S1: Site specific assumptions and corrections

We sampled large cliffs with several hundred meters diameter to understand the erosion patterns of large near-vertical headwalls. The unconventional geometries of our depth profiles (constrained by the local geometry of the tunnels sampled) call for some conceptual adjustments before we can compute TCN exposure ages and proceed to depth profile modelling.

First, the assumption of a *large, uniform sloped surface*^1^ is appropriate for an approximation of our sites as the sampled rock face is several times larger than the diameter of the relevant apparent attenuation length ($\Lambda_{f}$; for fast nucleonic particles in g cm^-2^), which corresponds to the length at which a particle intensity is attenuated by $e^{-1}$ upon penetrating a medium^2,3^. Specifically, for our sites the local surface needs to be a first order uniform circular plane with a minimum radius of $\geq2.75$ m $(\cong5*\Lambda_{f,e}\rho^{-1}$ for the largest apparent attenuation length; see supplement S2 and Table 1). We justify this approximation given the large headwall nature.

Second, all obstacles need to be opaque to incoming cosmic rays and they have to completely block the incoming particles. This mainly concerns the cosmic ray attenuation length in rock, which ranges between 0.32 and 0.5 m for fast neutrons^4^ and in the order of a few hundred m for most of the muon spectrum^5,6^. As the shielding of our sites is generated either by large mountains blocking the horizon, or the large headwall of the Eiger mountain itself, we are confident that all obstacles exceed this thickness by several orders of magnitude (with 10^2^ to 10^4^ m rock thickness).

Third, not all samples were collected perpendicular to the surface (Fig. S1). We accounted for this skewness through geometric considerations,

$z=z^{*}*\sin(\alpha+\beta)$, (S1.1)

where $z^{*}$ is the measured surface distance, $z$ corresponds to the true perpendicular distance to the sampled surface (see also Fig. S1), $\alpha$ is the dip/slope (in dip-direction) and $\beta$ is the plunging angle of the depth profile (Fig. S1). All depths reported are perpendicular (z) to the surface. This causes some samples at depth not being exactly centered below the same midpoint, but rather having an offset ($\Delta x$, $\Delta z$). This is considered through:

$\Delta z=z^{*}\cos(\alpha^{*})*\cos(90-\alpha)$ (S1.2)

and

$\Delta x=z^{*}\cos(\alpha^{*})*\sin(90-\alpha)$. (S1.3)

We then calculated depth below surface ($Z$; in g cm^-2^) related to the vertical distance ($z$):

$Z\left( z \right)=\int_{0}^{z} \rho\left( z \right)dz$. (S1.4)

Following assumption 1 as noted above, all samples still represent the same rock surface (with $\Delta x$ ranging between 0 and 0.62 m and $\Delta z$ between 0 and 1.83 m; see Table S2). Nuclide production is strongly dependent on the elevation/atmospheric pressure^2,3^ to which production rates are scaled. An elevation difference of 10 m can be translated to ~1 hPa difference in atmospheric pressure, which yields in an uncertainty of ~1% in production for sea level settings, decreasing with higher elevations^2,3,7,8^. Our largest $\Delta z$ is smaller than 2m and thus much smaller than the systematic uncertainty on our elevation (10 m) which is based on the DEM employed for this paper. Hence, this does not introduce any further uncertainties related to elevation; therefore it is not treated hereafter.

*
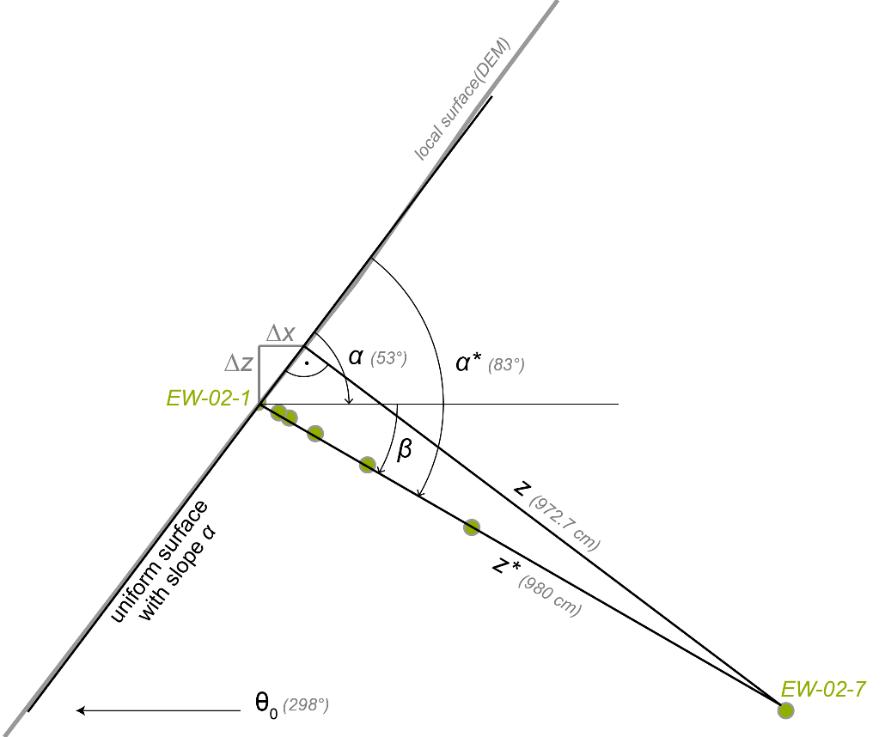
*

Figure S1: Sketch of general depth profile geometry (as discussed in the text) for profile EW-02 in dip-direction. All light quantities (green and grey) are site- and sample-specific (for EW-02-7).

# S2: Shielding calculation and evaluation

Depth profile sample geometry (see supplement S1) affects the production from spallation ($P_{s,m}$) as local topography and surface geometry (1) shields part of the incoming cosmic ray flux and (2) changes the apparent attenuation length for fast nucleonic particles (mostly neutrons), due to the inclination dependence of the incoming particle flux^1,2^. The nuclide production from the spallation of a TCN (e.g. ^36^Cl) is calculated in the following way^2,8–12^:

$P_{s,m}\left( Z \right)=S_{T}\sum_{k} S_{el,s}P_{m,k}\left( 0 \right)C_{k}\exp\left( -\frac{Z}{\Lambda_{f,e}} \right)$, (S2.1)

where $P_{m,k}(0)$ is the modern sea-level, high-latitude production rate for species m in element k, $S_{el,s}$ is the scaling factor for the specific reaction and $C_{k}$ is the concentration of the element. These account for the proportionality between the production related to the incoming flux and the concentration of the target element (detailed production equations can be found in the supplement of Marrero et al.^8^, based on Gosse & Phillips^2^). $S_{T}$ is usually calculated as the ratio between the total local unobstructed flux ($F_{max}$) intensity integrated over the complete sky $\Phi_{f}(max)$ and the actual surface flux ($F$) intensity integrated over the visible sky $\Phi_{f}\left( \phi,\theta\right)$:

$S_{T}=\frac{F}{F_{\max}}=\frac{\Phi_{f}\left( \phi,\theta\right)}{\Phi_{f}\left( max \right)}=\frac{\int_{\theta=0}^{2\pi} \int_{\phi=0}^{min\left\{ \begin{aligned} \phi_{s} \\ \phi_{t} \end{aligned} \right.} F\left( \phi\right)\sin\left( \phi\right)d\phi d\theta}{\Phi_{f}\left( max \right)}$. (S2.2)

Here, $\phi$ is the angle of inclination, $\theta$ the azimuth and $\phi_{t}$,$\phi_{s}$ are the min. inclination angles given by the topography or the slope geometry ^1,2^. The angular flux dependence $F(\phi)$ is implemented as

$F\left( \phi\right)= F_{0} {cos}^{m}(\phi)$ (S2.3)

^1–3,8,13^, where $F_{0}$corresponds to the maximum intensity and is a fitted value of $m=2.3\pm0.5$from^14^, despite a variety of values proposed in literature^11^. The open sky flux ($F_{max}$) is given by the analytical formula ^1^

$F_{max}=\frac{2\pi F_{0}}{m+1}$. (S2.4)

For a dipping surface, an angle $\gamma(\phi,\theta)$ is defined between the normal of the surface and an arbitrary incidence angle (Fig. S2). The normal is defined by a dipping angle $\alpha$ (= slope, dip) in the direction of dip $\theta_{0}$. Using the spherical law of cosines^2^ the geometrical relation of:

$\cos(\gamma\left( \phi,\theta\right)=\cos\left( \alpha\right)\cos(\phi)+\sin(\alpha)\sin(\phi)\cos(\theta_{0}-\theta)$ (S2.5)

can be used to relate the shielding induced by a dipping surface with the shielding of the surrounding topography.


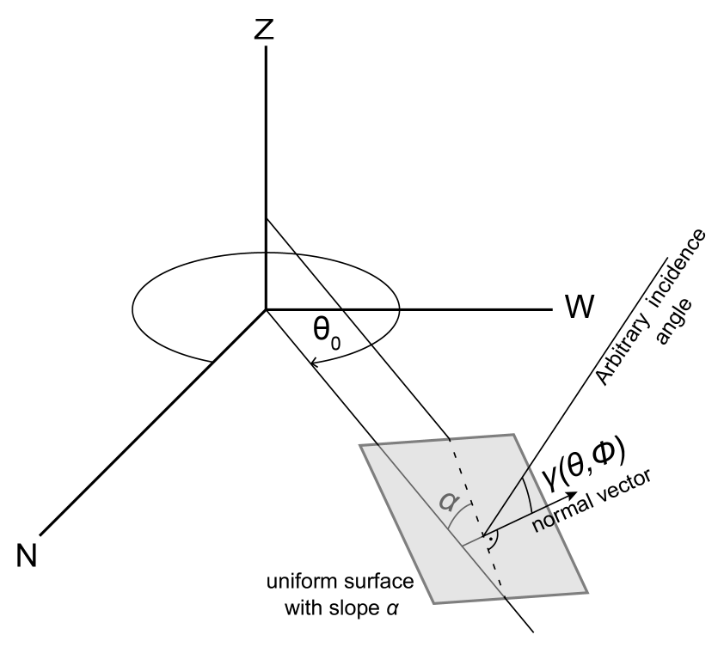


Figure S2: Illustration of the geometrical relations used in S2 (after Gosse and Phillips ^2^).

By combining equations (S2.2) - (S2.5) we end up with:

$S_{T}=\frac{\int_{\theta=0}^{2\pi} \int_{\phi=0}^{min\left\{ \begin{aligned} \phi_{s} \\ \phi_{t} \end{aligned} \right.} F_{0} {cos}^{m}\left( \phi\right)\sin\left( \phi\right)\left( \cos\left( \alpha\right)\cos\left( \phi\right)+\sin\left( \alpha\right)\sin\phi\cos\left( \theta_{0}-\theta\right) \right)d\phi d\theta}{\frac{2\pi F_{0}}{m+1}}$. (S2.6)

Second, the particle-specific and energy-dependent attenuation length governs the production rate with depth below a surface. It is defined as the flux-weighted integral of the attenuation length for fast nucleonic particles (>10MeV) over the entire sky. Usually it is implemented as apparent attenuation length ($\Lambda_{f}$) defined for a flat sample site with no topographic shielding, which is scaled to longitude, latitude and elevation (with often varying scaling models; see ^3,15–18^). An effective apparent attenuation length ($\Lambda_{f,e}$) can then be defined for a production at depth perpendicular to the surface^2^:

$S_{tot}=\frac{\Lambda_{f}}{\Lambda_{f,e}}S_{T}$, (S2.7)

where $S_{tot}$ is the total sample specific production scaling at depth perpendicular to a slope surface, accounting for topographic and geometric shielding and for a change in the effective attenuation length. By using the equations and geometrical relations from above $\Lambda_{f,e}$ is given by:

$\Lambda_{f,e}=\Lambda_{f}\frac{\int_{\theta=0}^{2\pi} \int_{\phi=0}^{\phi_{s}} F_{0} {cos}^{m}\left( \phi\right)\sin\left( \phi\right)\left( \cos\left( \alpha\right)\cos\left( \phi\right)+\sin\left( \alpha\right)\sin\phi\cos\left( \theta_{0}-\theta\right) \right)d\phi d\theta}{\int_{\theta=0}^{2\pi} \int_{\phi=0}^{\phi_{s}} F_{0} {cos}^{m}\left( \phi\right)\sin\left( \phi\right)d\phi d\theta}$ (S2.8)

This is a standard approach for the calculation of topographic shielding and effective fast neutron attenuation length^2^, and it is readily available through online calculation tools (i.e. the CRONUS-Earth Topographic shielding calculator, available at: <http://cronus.cosmogenicnuclides.rocks/2.0>). We employed the online CRONUS-Earth Topographic shielding calculator to calculate shielding ($S_{T}$, $\Lambda_{f,e}$) corrections (supplement table S1). We note that the two effects (and thus parameters) are not necessarily independent of each other^1^ and thus should be calculated for complex settings (as is ours) as a total flux scaling factor ($S_{T,D}$; after ^1^) for each sample:

$S_{T,D}(Z,\alpha)=\frac{F}{F_{max}}$. (S2.9)

In principle, they yield a similar, yet slightly different way, to calculate such a shielding factor (by incorporating the scaling for depth already).

The actual flux can also be expressed as^19^:

$F=\frac{\int_{\theta=0}^{2\pi} \int_{max\left\{ \begin{aligned} \phi_{t} \\ \phi_{s} \end{aligned} \right.}^{\pi/2} F_{0} {sin}^{m}\left( \phi\right)e^{-d/\Lambda}\cos(\phi)d\phi d\theta}{\frac{2\pi F_{0}}{m+1}}$. (S2.10)

Thus, the total shielding is given by

$S_{T,D}\left( Z,\alpha\right)=\frac{\int_{\theta=0}^{2\pi} \int_{max\left\{ \begin{aligned} \phi_{t} \\ \phi_{s} \end{aligned} \right.}^{\pi/2} {sin}^{m}\left( \phi\right)e^{-d/\Lambda}\cos(\phi)d\phi d\theta}{\frac{2\pi}{m+1}}$, (2.11)

with $\Lambda$ as the true particle attenuation length^1,2,19,20^, and $d$ being the distance to the dipping surface, expressed as

$d(\phi,\theta)=\frac{Z}{\cos\left( \alpha\right)\sin\left( \phi\right)+\sin\left( \alpha\right)cos( \phi)cos( \theta-\theta_{d})}$. (2.11)

The lower limit of the sloped surface for the integral is given by

$\phi_{s}=\left| {-arctan(\left( \cos\theta-\theta_{d})\tan\left( \alpha\right) \right), \frac{\pi}{2}\leq\theta-\theta_{d}<\frac{3\pi}{2} \atop0, -\frac{\pi}{2}\leq\theta-\theta_{d}<\frac{\pi}{2}} \right.$ (2.12)

and the true particle attenuation length ($\Lambda$) is given by

$\Lambda=\frac{m+2}{m+1}\Lambda_{f}$ (2.13)

as a relation to the apparent attenuation length^2,19^.

We calculated $S_{T}$ after Gosse and Phillips^2^ with the use of equations (S2.6) – (S2.8) for each sample (using the effective apparent attenuation length for depth propagation). We solved equations (S2.10) – (S2.13) numerically using a trapezoidal integration scheme for $S_{T,D}$ after Dunne et al.^1^ to evaluate the dependence of the topographic/geometric shielding and change in effective attenuation length for production by spallation of fast particles (see table S2). We did this to demonstrate that for our study both, $S_{T}$ and $S_{T,D}$, are similar enough (within maximum difference of 5%) to be treated as independent. Production from thermal and epithermal neutrons is calculated differently for our purpose^8^. Therefore the approach following Lal (1991)^2^ for shielding correction is justified, and site-specific shielding can be used to account for topographic blocking on the hadronic flux and the muon flux. Effective attenuation lengths for fast particles can be used for correcting effects of the slope geometry on the flux composition (i.e., flux “hardening or softening”). Conveniently, these are standard inputs for the used software.

Depth profile geometry and the geometry of the large cliffs we sampled also affect the TCN production from muons. The cosmic ray muon flux is different from the hadronic flux as muons have much lower interaction rates and thus penetrate much further into rock. Additionally the low energy muons are absorbed rapidly by muon capture leading to an average energy increase for muons with depth. Therefore, the muon flux at depth cannot be described by a mean attenuation length (as can be the hadronic flux). As a result, common calculations for muogenic production^3,8^ calculate two production pathways using experimentally derived equations^21,22^. First, the production from fast muons is given by

$P_{\mu,f}=S_{T}\phi_{\mu,tot}(Z)\beta(Z)({\overline{E}(Z))}^{\alpha}\sigma_{0}N_{a,i}$, (2.14)

where $\overline{E}$ is the mean muon energy at depth, $N_{a,i}$ the number density of atoms in the target element, $\phi_{\mu,tot}$ the total, site scaled muon flux, $\beta$ an attenuation function, $\sigma_{0}$ the cross-section at 1 GeV, and $\alpha$ a coefficient for the energy dependence of the cross section.

Second, the production by negative muon capture, is given by

$P_{\mu-}=S_{T}*R_{\mu-}\left( Z \right)f_{i,c}f_{i,D}f_{i}^{*}$, (2.15)

where $R_{\mu-}$ is the negative muon stopping power at depth, $f_{i,c}$ is the nuclide specific compound factor, $f_{i,D}$ the nuclide specific probability of not decaying before entering the nucleus core and $f_{i}^{*}$ the particle emission channel probability. Both production pathways need a shielding correction ($S_{T}$), as our rock cliff are much thicker than 400 m and thus, effectively block all of the incoming muons from obstructed parts of the sky. We used the shielding factor already calculated for spallation, due to the similar angular dependence of the muogenic and hadronic flux (see Eq. S2.3$)$with an empirically derived exponential function or constant (i.e., $m$). For the elevation range of our study the muogenic angular dependence (~2.1) is very close to the used value of 2.3 for the spallogenic production, therefore we employed the same shielding correction factor ($S_{T}$) for correcting the surface muon flux. The calculation of the muon flux at depth further included empirically derived muon attenuation equations^21,22^.

| **Sample** | **profile distance**  $\boldsymbol{z}^{\mathbf{*}}$ | **sample surface**  $\boldsymbol{z}$ | **mass depth**  $\boldsymbol{Z}$ | **delta elev.**  **Δz** | **Total sample shielding^a^**  $\boldsymbol{S}_{\boldsymbol{T}}$ | **Total sample shielding^b^**  $\boldsymbol{S}_{\boldsymbol{T}\mathbf{,}\boldsymbol{D}}$ | **Difference**  $\boldsymbol{\Delta S}$ |
| --- | --- | --- | --- | --- | --- | --- | --- |
|  | cm | cm | g cm^-2^ | cm |  |  |  |
| EM-01-1 | 0 | 0 | 0 | 0 | 0.64 | 0.64 | 0.00 |
| EM-01-2 | 20 | 19.7 | 52.8 | 3.4 | 0.37 | 0.32 | 0.05 |
| EM-01-3 | 65 | 64.0 | 171.4 | 11.1 | 0.10 | 0.11 | 0.00 |
| EM-01-4 | 115 | 113.2 | 303.3 | 19.7 | 0.02 | 0.04 | -0.01 |
| EM-01-5 | 195 | 191.9 | 514.3 | 33.3 | 0.00 | 0.01 | -0.01 |
| EM-01-6 | 300 | 295.3 | 791.3 | 51.3 | 0.00 | 0.00 | 0.00 |
| EM-01-7 | 900 | 885.8 | 2373.9 | 153.8 | 0.00 | 0.00 | 0.00 |
| EM-02-1 | 0 | 0 | 0 | 0 | 0.58 | 0.58 | 0.00 |
| EM-02-2 | 25 | 25 | 64.6 | 6.4 | 0.30 | 0.26 | 0.04 |
| EM-02-3 | 70 | 70 | 180.8 | 18.0 | 0.09 | 0.10 | 0.00 |
| EM-02-4 | 125 | 125 | 322.8 | 32.2 | 0.02 | 0.03 | -0.01 |
| EM-02-5 | 200 | 200 | 516.5 | 51.5 | 0.00 | 0.01 | -0.01 |
| EM-02-6 | 300 | 300 | 774.8 | 77.3 | 0.00 | 0.00 | 0.00 |
| EM-02-7 | 710 | 710 | 1833.6 | 182.8 | 0.00 | 0.00 | 0.00 |
| EM-03-1 | 0 | 0 | 0 | 0 | 0.58 | 0.59 | 0.00 |
| EM-03-2 | 20 | 20 | 52.9 | 3.1 | 0.32 | 0.27 | 0.05 |
| EM-03-3 | 75 | 75 | 198.5 | 11.7 | 0.06 | 0.07 | -0.01 |
| EM-03-4 | 120 | 120 | 317.6 | 18.7 | 0.01 | 0.03 | -0.01 |
| EM-03-5 | 200 | 200 | 529.3 | 31.2 | 0.00 | 0.01 | 0.00 |
| EM-03-6 | 300 | 300 | 793.9 | 46.9 | 0.00 | 0.00 | 0.00 |
| EM-03-7 | 875 | 875 | 2315.5 | 136.6 | 0.00 | 0.00 | 0.00 |
| EW-02-1 | 0 | 0 | 0 | 0 | 0.49 | 0.49 | 0.00 |
| EW-02-2 | 30 | 29.8 | 79.8 | 2.5 | 0.28 | 0.27 | 0.01 |
| EW-02-3 | 50 | 49.6 | 133.0 | 4.2 | 0.20 | 0.19 | 0.01 |
| EW-02-4 | 100 | 99.3 | 266.0 | 8.3 | 0.08 | 0.08 | 0.00 |
| EW-02-5 | 200 | 198.5 | 532.0 | 16.7 | 0.01 | 0.02 | 0.00 |
| EW-02-6 | 400 | 397.0 | 1064.0 | 33.4 | 0.00 | 0.00 | 0.00 |
| EW-02-7 | 980 | 972.7 | 2606.8 | 81.8 | 0.00 | 0.00 | 0.00 |
| EW-03-1 | 0 | 0 | 0 | 0 | 0.45 | 0.45 | 0.00 |
| EW-03-2 | 40 | 39.9 | 107.0 | 2.2 | 0.14 | 0.13 | 0.01 |
| EW-03-3 | 75 | 74.9 | 200.7 | 4.2 | 0.05 | 0.06 | 0.00 |
| EW-03-4 | 175 | 174.7 | 468.3 | 9.7 | 0.00 | 0.01 | 0.00 |
| EW-03-5 | 250 | 249.6 | 669.0 | 13.9 | 0.00 | 0.00 | 0.00 |
| EW-03-6 | 500 | 499.2 | 1337.9 | 27.7 | 0.00 | 0.00 | 0.00 |
| EW-03-7 | 900 | 898.6 | 2408.2 | 49.9 | 0.00 | 0.00 | 0.00 |
| ***^*^****for fast particles; ^a^after* Gosse and Phillips (2001)*; ^b^after* Dunne et al. (1999) | | | | | | |  |

Table S2: Sample depth and corresponding shielding parameters, for discussion see supplement S2.

# S3: Sample chemistry

| **Major element** | **SiO_2_** | **Al_2_O_3_** | **Fe_2_O_3_ total** | **MnO** | **MgO** | **CaO** | **Na_2_O** | **K_2_O** | **TiO_2_** | **P_2_O_5_** | | **Gd** | **SM** | **Th** | **U** | | **B** | |
| --- | --- | --- | --- | --- | --- | --- | --- | --- | --- | --- | --- | --- | --- | --- | --- | --- | --- | --- |
| Analysis Method | FUS-ICP | | | | | | | | | | FUS-MS | | | | | PGNAA | |  |
| Unit | % | % | % | % | % | % | % | % | % | % | | ppm | ppm | ppm | ppm | | ppm | |
| Detection Limit | 0.01 | 0.01 | 0.01 | 0.001 | 0.01 | 0.01 | 0.01 | 0.01 | 0.001 | 0.01 | | 0.1 | 0.1 | 0.1 | 0.1 | | 2 | |
| **EM-01-1*** | 1.20 | 0.4 | 0.11 | 0.01 | 0.48 | 52.33 | n.d. | 0.12 | <0.001 | n.d. | | 0.8 | 0.6 | 0.2 | 0.5 | | <10 | |
| EM-01-2 | 1.18 | 0.31 | 0.19 | 0.013 | 0.5 | 54.9 | 0.05 | 0.05 | 0.015 | 0.02 | | 0.7 | 0.5 | 0.2 | 0.3 | | 5 | |
| EM-01-3 | 0.82 | 0.2 | 0.12 | 0.012 | 0.46 | 54.89 | 0.03 | 0.04 | 0.007 | 0.04 | | 0.6 | 0.5 | 0.2 | 0.4 | | < 2 | |
| EM-01-4 | 0.83 | 0.22 | 0.14 | 0.013 | 0.47 | 54.94 | 0.04 | 0.04 | 0.007 | 0.04 | | 0.7 | 0.6 | 0.2 | 0.3 | | 8 | |
| EM-01-5 | 1.08 | 0.33 | 0.14 | 0.012 | 0.49 | 54.5 | 0.04 | 0.06 | 0.01 | 0.03 | | 0.8 | 0.7 | 0.3 | 0.3 | | < 2 | |
| EM-01-6 | 1 | 0.33 | 0.13 | 0.012 | 0.48 | 55.15 | 0.05 | 0.06 | 0.009 | 0.03 | | 0.7 | 0.6 | 0.2 | 0.3 | | < 2 | |
| EM-01-7 | 0.7 | 0.22 | 0.08 | 0.01 | 0.45 | 55.5 | 0.03 | 0.04 | 0.006 | 0.04 | | 0.7 | 0.5 | 0.2 | 0.4 | | 2 | |
| EM-02-1 | 1.16 | 0.33 | 0.16 | 0.016 | 0.5 | 54.54 | 0.05 | 0.06 | 0.009 | 0.04 | | 0.8 | 0.6 | 0.2 | 0.4 | | 5 | |
| EM-02-2 | 3.91 | 1.04 | 0.31 | 0.016 | 0.62 | 52.51 | 0.09 | 0.19 | 0.038 | 0.05 | | 1 | 1 | 0.7 | 0.4 | | 21 | |
| EM-02-3 | 0.97 | 0.28 | 0.14 | 0.013 | 0.52 | 54.58 | 0.04 | 0.05 | 0.009 | 0.03 | | 0.8 | 0.6 | 0.2 | 0.4 | | < 2 | |
| EM-02-4 | 1.14 | 0.34 | 0.18 | 0.013 | 0.52 | 54.04 | 0.04 | 0.07 | 0.012 | 0.04 | | 0.7 | 0.7 | 0.3 | 0.3 | | 5 | |
| EM-02-5 | 1.43 | 0.39 | 0.22 | 0.015 | 0.6 | 53.81 | 0.05 | 0.07 | 0.013 | 0.04 | | 0.9 | 0.7 | 0.3 | 0.3 | | < 2 | |
| EM-02-6 | 0.6 | 0.15 | 0.07 | 0.011 | 0.44 | 55.78 | 0.02 | 0.03 | 0.005 | 0.03 | | 0.6 | 0.4 | 0.1 | 0.3 | | < 2 | |
| EM-02-7 | 1.07 | 0.27 | 0.13 | 0.011 | 0.44 | 55.3 | 0.07 | 0.05 | 0.005 | 0.03 | | 0.5 | 0.4 | 0.1 | 0.3 | | < 2 | |
| EM-03-1 | 0.7 | 0.18 | 0.07 | 0.013 | 0.49 | 55.22 | 0.03 | 0.04 | 0.006 | 0.04 | | 0.6 | 0.5 | 0.2 | 0.2 | | < 2 | |
| EM-03-2 | 1.01 | 0.32 | 0.13 | 0.013 | 0.49 | 54.36 | 0.06 | 0.07 | 0.008 | 0.04 | | 0.8 | 0.6 | 0.3 | 0.2 | | 4 | |
| EM-03-3 | 0.39 | 0.1 | 0.05 | 0.013 | 0.46 | 55.85 | 0.02 | 0.02 | 0.004 | 0.04 | | 0.7 | 0.6 | 0.1 | 0.2 | | < 2 | |
| EM-03-4 | 0.54 | 0.19 | 0.11 | 0.013 | 0.51 | 55.89 | 0.04 | 0.03 | 0.006 | 0.03 | | 0.7 | 0.5 | 0.2 | 0.4 | | < 2 | |
| EM-03-5 | 0.46 | 0.16 | 0.07 | 0.013 | 0.5 | 54.88 | 0.03 | 0.03 | 0.005 | 0.05 | | 0.7 | 0.5 | 0.2 | 0.3 | | < 2 | |
| EM-03-6 | 0.77 | 0.21 | 0.09 | 0.013 | 0.46 | 54.61 | 0.04 | 0.04 | 0.006 | 0.03 | | 0.6 | 0.6 | 0.2 | 0.3 | | 11 | |
| EM-03-7 | 0.56 | 0.13 | 0.09 | 0.012 | 0.49 | 55.64 | 0.02 | 0.03 | 0.004 | 0.03 | | 0.7 | 0.5 | 0.1 | 0.3 | | < 2 | |
| EW-02-1 | 21.19 | 1.57 | 1.35 | 0.007 | 0.68 | 37.57 | 0.06 | 0.08 | 0.108 | 0.03 | | 1.7 | 1.9 | 2.2 | 1.2 | | 25 | |
| EW-02-2 | 25.01 | 1.51 | 1.27 | 0.007 | 0.56 | 38.33 | 0.08 | 0.21 | 0.126 | 0.02 | | 1.4 | 1.6 | 2.4 | 1.3 | | 20 | |
| EW-02-3 | 20.72 | 1.54 | 1.57 | 0.009 | 0.56 | 42.46 | 0.1 | 0.21 | 0.114 | 0.02 | | 1.9 | 2.1 | 2.3 | 1.2 | | 13 | |
| EW-02-4 | 2.25 | 0.33 | 0.25 | 0.007 | 0.34 | 53.64 | 0.05 | 0.05 | 0.014 | < 0.01 | | 0.2 | 0.2 | 0.3 | 0.9 | | < 2 | |
| EW-02-5 | 1.47 | 0.41 | 0.3 | 0.006 | 0.46 | 54.23 | 0.05 | 0.08 | 0.015 | < 0.01 | | 0.2 | 0.2 | 0.3 | 1.3 | | 3 | |
| EW-02-6 | 1.72 | 0.52 | 0.36 | 0.006 | 0.45 | 54.19 | 0.04 | 0.09 | 0.02 | 0.02 | | 0.4 | 0.4 | 0.4 | 1.1 | | < 2 | |
| EW-02-7 | 3.35 | 0.27 | 0.23 | 0.006 | 0.35 | 52.99 | 0.04 | 0.05 | 0.008 | 0.01 | | 0.1 | 0.2 | 0.2 | 0.9 | | < 2 | |
| EW-03-1 | 3.57 | 0.48 | 0.54 | 0.009 | 0.24 | 53.05 | 0.04 | 0.09 | 0.019 | 0.01 | | 0.4 | 0.4 | 0.5 | 0.6 | | 5 | |
| EW-03-2 | 1.91 | 0.39 | 0.59 | 0.009 | 0.23 | 53.87 | 0.05 | 0.07 | 0.012 | 0.02 | | 0.3 | 0.4 | 0.4 | 0.6 | | 9 | |
| EW-03-3 | 2.12 | 0.41 | 0.77 | 0.009 | 0.23 | 54.38 | 0.04 | 0.07 | 0.015 | < 0.01 | | 0.4 | 0.4 | 0.5 | 0.5 | | < 2 | |
| EW-03-4 | 1.4 | 0.37 | 0.44 | 0.009 | 0.22 | 54.06 | 0.05 | 0.07 | 0.011 | < 0.01 | | 0.3 | 0.3 | 0.3 | 0.6 | | 7 | |
| EW-03-5 | 1.81 | 0.29 | 0.65 | 0.01 | 0.2 | 54.11 | 0.03 | 0.05 | 0.01 | < 0.01 | | 0.2 | 0.2 | 0.3 | 0.5 | | 4 | |
| EW-03-6 | 5.72 | 0.79 | 0.89 | 0.012 | 0.24 | 51.15 | 0.07 | 0.15 | 0.03 | 0.01 | | 0.7 | 0.7 | 0.8 | 0.6 | | 4 | |
| EW-03-7 | 5.52 | 0.78 | 1.1 | 0.011 | 0.28 | 51.27 | 0.05 | 0.14 | 0.037 | 0.03 | | 0.9 | 1 | 0.9 | 0.6 | | 5 | |

Table S3: Chemical data for the analyzed limestone samples (*Sample EM-01-1 was measured using FUS-MS on samples oxidized with sodium peroxide, Na_2_O_2_); n.d. = not determined.

# S4: Rock density estimation

A total of 16 samples were taken, underground in the railway tunnel and at the surface (coordinates given in table S4), to determine density properties (and variability thereof) of the sampled limestone units. These consist of upper Jurassic, originally micritic and completely recrystallized limestone and lower Cretaceous, chert-rich limestone, also recrystallized under the same conditions^23^. The collected sample mass was usually >1 kg, to yield sufficient material to prepare representative powdered and solid splits, as well as spare samples. Bulk densities were determined by volume measurements of water-suspended samples coated with paraffin wax^24^. Coating was necessary due to the presence of connected porosity and the resulting degassing of the samples in different auxiliary liquids (e.g. different paraffin oils). Grain density was determined on powdered sample aliquots (~10g), using an AccuPyc 1340 He-pycnometer, capable for measuring volumes of dried samples at high precision^25^. Porosity was calculated form bulk and grain density^26^. Values given in table S4 are averages of at least 5 (3 for bulk density) individual measurements. Reported uncertainties (1 sigma) include propagated errors that account both for accuracy and for precision. The results are consistent for all samples, giving values of 2.68 ± 0.02 gcm^-3^, 2.74 ± 0.02 gcm^-3^ and 1.8 ± 0.5% for bulk density, grain density and porosity respectively.

| **Sample** | **grain density**  **[gcm^-3^]** | | | **bulk density**  **[gcm^-3^]** | | | **porosity [%]** | | **X**  **[m]** | | **Y**  **[m]** | **Elev.**  **[m]** |
| --- | --- | --- | --- | --- | --- | --- | --- | --- | --- | --- | --- | --- |
| AM-04 | 2.73 | ± | 0.007 | 2.67 | ± | 0.01 | | 1.43 | | 643850 | 157952 | 3129 |
| JT-10 | 2.74 | ± | 0.008 | 2.69 | ± | 0.02 | | 1.23 | | 643089 | 156945 | 3243 |
| T-11 | 2.73 | ± | 0.007 | 2.66 | ± | 0.02 | | 1.91 | | 643792 | 158357 | 3105 |
| JT-12 | 2.73 | ± | 0.008 | 2.64 | ± | 0.02 | | 2.53 | | 643832 | 158094 | 3158 |
| JT-13 | 2.72 | ± | 0.006 | 2.66 | ± | 0.01 | | 1.69 | | 643832 | 158094 | 3158 |
| JT-14 | 2.73 | ± | 0.003 | 2.68 | ± | 0.02 | | 1.68 | | 643347 | 157289 | 3217 |
| JT-15 | 2.73 | ± | 0.004 | 2.68 | ± | 0.02 | | 1.65 | | 643351 | 157295 | 3216 |
| JT-16 | 2.74 | ± | 0.004 | 2.69 | ± | 0.01 | | 1.84 | | 643351 | 157296 | 3216 |
| JT-17 | 2.74 | ± | 0.005 | 2.66 | ± | 0.02 | | 2.84 | | 643358 | 157303 | 3216 |
| GH-01 | 2.72 | ± | 0.004 | 2.63 | ± | 0.02 | | 2.96 | | 641095 | 156976 | 2798 |
| JT-30 | 2.72 | ± | 0.004 | 2.65 | ± | 0.03 | | 2.27 | | 643820 | 158065 | 3160 |
| JT-31 | 2.73 | ± | 0.004 | 2.68 | ± | 0.02 | | 1.19 | | 643820 | 158065 | 3160 |
| JT-32 | 2.75 | ± | 0.003 | 2.69 | ± | 0.02 | | 1.04 | | 643190 | 157082 | 3232 |
| JT-33 | 2.80 | ± | 0.004 | 2.71 | ± | 0.02 | | 2.09 | | 643190 | 157082 | 3232 |
| JT-34 | 2.82 | ± | 0.004 | 2.75 | ± | 0.03 | | 1.99 | | 643370 | 157321 | 3213 |
| JT-35 | 2.75 | ± | 0.003 | 2.69 | ± | 0.02 | | 1.12 | | 643425 | 157395 | 3208 |

Table S4: Samples used for density estimation (locations given in Swiss Coordinates, CH-1903).

# S5: Apparent surface exposure ages

Apparent exposure ages were calculated for all samples (not only surface samples) following the assumptions given in the methods section. Results can be found in the zip archive (“S5: Apparent surface exposure ages”) as Excel^TM^ sheet (“Results.xlsx”) with results including path specific production and total production. Further, we provide the complete input sheet used for the CRONUScalc web calculator (“Input.xlsx”).

# S6: Monte Carlo input & results

This supplementary zip file contains the Mathcad^TM^ code used for the Monte Carlo depth profile modelling as well as necessary input files. We further provide all the Excel^TM^ input files used for the calculations as well as the run-specific MC model setup as html files (containing all input parameters) and the MC raw results used for discussions and figures in the main text. The solution space for erosion rate and exposure age for all model runs is dominated by the chosen initial values, most notable the net erosion cut-off (Fig. S6). Notable changes to the original Mathcad^TM^ code^27^ include the usage of the shielding correction for the muon production and an increase in resolution of the muon depth fit. Full documentation is provided in an additional html file.


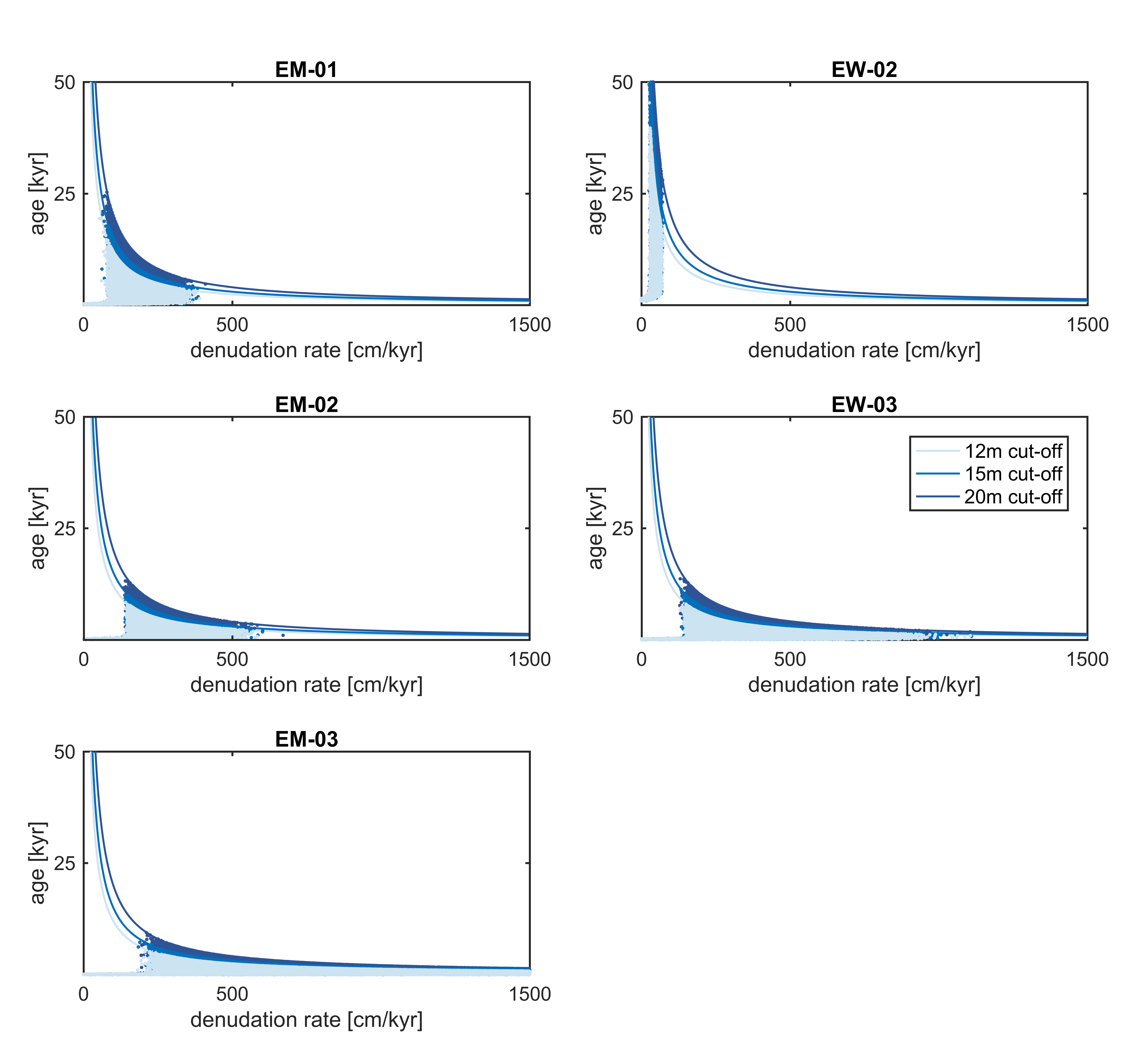


Figure S6: Modelled profile ages plotted against erosion rate with indicated profile-specific confidence interval for three different net erosion cut-offs. Single points represent the model run specific, color-coded solution. The range of the erosion rate estimation remains constant for different cut-offs illustrating the robustness of the parameter estimation.

# Supplement References

1. Dunne, J., Elmore, D. & Muzikar, P. Scaling factors for the rates of production of cosmogenic nuclides for geometric shielding and attenuation at depth on sloped surfaces. *Geomorphology* **27**, 3–11 (1999).

2. Gosse, J. C. & Phillips, F. M. Terrestrial in situ cosmogenic nuclides: Theory and application. *Quat. Sci. Rev.* **20**, 1475–1560 (2001).

3. Balco, G., Stone, J. O., Lifton, N. A. & Dunai, T. J. A complete and easily accessible means of calculating surface exposure ages or erosion rates from 10Be and 26Al measurements. *Quat. Geochronol.* **3**, 174–195 (2008).

4. Balco, G. Simple computer code for estimating cosmic-ray shielding by oddly shaped objects. *Quat. Geochronol.* **22**, 175–182 (2014).

5. Braucher, R., Brown, E. T., Bourlès, D. L. & Colin, F. In situ produced 10Be measurements at great depths: Implications for production rates by fast muons. *Earth Planet. Sci. Lett.* **211**, 251–258 (2003).

6. Balco, G. Production rate calculations for cosmic-ray-muon-produced10Be and26Al benchmarked against geological calibration data. *Quat. Geochronol.* **39**, 150–173 (2017).

7. Dunai, T. J. Influence of secular variation of the geomagnetic field on production rates of in situ produced cosmogenic nuclides. *Earth Planet. Sci. Lett.* **193**, 197–212 (2001).

8. Marrero, S. M. *et al.* Cosmogenic nuclide systematics and the CRONUScalc program. *Quat. Geochronol.* **31**, 160–187 (2016).

9. Marrero, S. M., Phillips, F. M., Caffee, M. W. & Gosse, J. C. CRONUS-Earth cosmogenic 36Cl calibration. *Quat. Geochronol.* **31**, 199–219 (2016).

10. Schimmelpfennig, I. *et al.* Sources of in-situ36Cl in basaltic rocks. Implications for calibration of production rates. *Quat. Geochronol.* **4**, 441–461 (2009).

11. Dunai, T. J. *Cosmogenic Nuclides*. *Cambridge University Press* **36**, (Cambridge University Press, 2010).

12. Alfimov, V. & Ivy-Ochs, S. How well do we understand production of 36Cl in limestone and dolomite? *Quat. Geochronol.* **4**, 462–474 (2009).

13. Heidbreder, E., Pinkau, K., Reppin, C. & Schönfelder, V. Measurements of the distribution in energy and angle of high-energy neutrons in the lower atmosphere. *J. Geophys. Res.* **76**, 2905–2916 (1971).

14. Nishiizumi, K. *et al.* Cosmic ray production rates of 10 Be and 26 Al in quartz from glacially polished rocks. *J. Geophys. Res.* **94**, 17907 (1989).

15. Lifton, N., Sato, T. & Dunai, T. J. Scaling in situ cosmogenic nuclide production rates using analytical approximations to atmospheric cosmic-ray fluxes. *Earth Planet. Sci. Lett.* **386**, 149–160 (2014).

16. Martin, L. C. P. *et al.* The CREp program and the ICE-D production rate calibration database: A fully parameterizable and updated online tool to compute cosmic-ray exposure ages. *Quat. Geochronol.* **38**, 25–49 (2017).

17. Phillips, F. M., Hinz, M., Marrero, S. M. & Nishiizumi, K. Cosmogenic nuclide data sets from the Sierra Nevada, California, for assessment of nuclide production models: II. Sample sites and evaluation. *Quat. Geochronol.* **35**, 101–118 (2016).

18. Stone, J. O. H., Evans, J. M., Fifield, L. K., Allan, G. L. & Cresswell, R. G. Cosmogenic Chlorine-36 Production in Calcite by Muons. *Geochim. Cosmochim. Acta* **62**, 433–454 (1998).

19. Tikhomirov, D., Akçar, N., Ivy-Ochs, S., Alfimov, V. & Schlüchter, C. Calculation of shielding factors for production of cosmogenic nuclides in fault scarps. *Quat. Geochronol.* **19**, 181–193 (2014).

20. Stone, J. O., Allan, G. L., Fifield, L. K. & Cresswell, R. G. Cosmogenic chlorine-36 from calcium spallation. *Geochim. Cosmochim. Acta* **60**, 679–692 (1996).

21. Heisinger, B. *et al.* Production of selected cosmogenic radionuclides by muons: 1. Fast muons. *Earth Planet. Sci. Lett.* **200**, 345–355 (2002).

22. Heisinger, B. *et al.* Production of selected cosmogenic radionuclides by muons: 2. Capture of negative muons. *Earth Planet. Sci. Lett.* **200**, 357–369 (2002).

23. Mair, D., Lechmann, A., Herwegh, M., Nibourel, L. & Schlunegger, F. Linking Alpine deformation in the Aar Massif basement and its cover units - The case of the Jungfrau-Eiger mountains (Central Alps, Switzerland). *Solid Earth* **9**, 1099–1122 (2018).

24. Blake, G. R. & Hartge, K. H. Bulk Density. in *Methods of Soil Analysis: Part 1—Physical and Mineralogical Methods, SSSA Book Series 5.1* (ed. Klute, A.) **9**, 363–375 (Soil Science Society of America, American Society of Agronomy, 1986).

25. Viana, M., Jouannin, P., Pontier, C. & Chulia, D. About pycnometric density measurements. *Talanta* **57**, 583–593 (2002).

26. Anovitz, L. M. & Cole, D. R. Characterization and Analysis of Porosity and Pore Structures. *Rev. Mineral. Geochemistry* **80**, 61–164 (2015).

27. Hidy, A. J., Gosse, J. C., Pederson, J. L., Mattern, J. P. & Finkel, R. C. A geologically constrained Monte Carlo approach to modeling exposure ages from profiles of cosmogenic nuclides: An example from Lees Ferry, Arizona. *Geochemistry, Geophys. Geosystems* **11**, (2010).
