# Supplementary material for: Fast long-term denudation rate of steep alpine headwalls inferred from cosmogenic 36Cl depth profiles
Source: Sci Rep. 2019 Jul 30;9:11023. doi: 10.1038/s41598-019-46969-0 (PMC6667707; doi:10.1038/s41598-019-46969-0)
Supplement: Supplementary file 2 — Supplementary Dataset [file 41598_2019_46969_MOESM2_ESM.zip › S6 Monte Carlo input & results/EM02_mod_Rev3_20_full.html]

 

**36****Cl depth profile simulator**

version 1.0

Site specific information:

**location:**

latitude (deg)

xx.xxx

longitude (deg)

xxx.xxx

altitude (m)

xxxx

**topographic/geometric shielding:**

The topographic shielding factor is 1 (no effect) as a default. If shielding has been measured, then an ascii text or Excel file can be loaded into the worksheet. To do this, right click on the disk icon below and select "Properties". From here you may specify your file type and path.This file should have two columns of data. Column 1 should contain a list of angular measurements of the horizon; column 2 should contain a list of azimuths associated with each horizon measurement. The program then makes a linear interpolation of the horizon based on these values and calculates a shielding factor. Additional shielding from samples collected on a dipping surface can be included if the strike and dip of the surface has been measured. If you already know your net topographic shielding factor, enter the value in the "define" field.

dipping surface?

strike (0 - 360 deg)

dip (0-90 deg; right hand rule)

horizon

or, define factor

(fraction)

Calculated or imputed shielding factor:

**cover (e.g. snow, loess etc.):**

This is your estimate of the percent change in production rate due to assumed periodic cover. Default is 1 (no effect).

cover factor (fraction)

Profile Data:

You may load your sample data from a text or Excel file rather than using the manual inputs above. To do this, first select the "file input" option below and then right click on the disk icon and select "Properties". From here you may specify your file type and path. Your data should be formatted as follows. Column order: sample depths, sample thicknesses, sample concentrations, 1σ measurement error. They should be in the same units as described above. The rows should should be ordered by sample depth (from shallowest to deepest).

Number of samples in profile

Samples and soil data

Water content

%

**Neutron**

**Neutron**

**Neutron**

**Neutron**

Atomic

mass

log

decrement

neutron

elastic c-s

thermal

absorbtion c-s

resonance

integral

**Muon**

**Muon**

**Muon**

**U & Th neutrons**

**U & Th neutrons**

capture prob

relative to oxygen

No decay in K-shell prob

Neutron yield after μ- capture

Stopping power

of element

Neutron yield

for U

Neutron yield

for Th

Molar percentage of each element in sample (mol/g):

Mass part of element in the soil accounting into water content

Molar percentage of each element in soil (mol/g):

Molar percentage of each element in air (mol/g):

**depths of samples, cm**

**thicknesses of samples, cm**

**36Cl concentrations of samples, 10^6 at/g**

**1****s** **total measurement errors of** **36Cl concentrations** **, 10^6 at/g**

**Elemental composition, fraction**

**Soil**

**Samples**

**Bulk density:**

You may vary bulk density with depth by entering parameters for a step function describing measured or assumed changes in density with depth (e.g., if you measured density to be 2.1 +/- 0.2 g cm-3 between 0-30 cm, 2.3 +/- 0.1 g cm-3 between 30-60 cm, and 2.4 +/- 0.2 g cm-3 at depths greater than 60 cm, then you would enter the numbers 0 30 60--space delimited--in the "depths" field, the numbers 2.1 2.3 2.4 in the "densities" field, and the numbers 0.2 0.1 0.2 in the "density errors" field). You may also treat bulk density as a constant with depth; you can chose either a random or normal distribution for this constant value. As a check, your depth function is displayed in the plots below.

Constant density with depth; values varies randomly or

normally:

Measured values for densities at different depths; values are normally distributed (do not use for constant density with depth):

**low**

(g cm-3)

**depths**

(cm)

**high**

(g cm-3)

**densities**

(g cm-3)

**1****s** **error**

(g cm-3)

**1****s** **density errors**

(g cm-3)

**Monte Carlo error simulation for bulk density at each sample--integrated over sample thickness**

depths of interest:

input data:

number of values:

LL and ff(x) create v cumulative density curves from normally distributed input data

step(x) assembles the inputed density step function (for display)

int(x) creates a cumulative mass curve from the inputed density step function (for display)

bulk density evaluted at each depth and integrated over thickness

mean and standard deviation of simulation

Production by muons:

This scheme follows the theoretical equations of Heisinger et al. 2002a, 2002b for production of muons vs depth. The approach of Balco 2008 is adopted to generate production rates via negative muon capture and fast muons at any given depth and altitude. A two-term exponential best fit for fast muons and a three-term exponential best fit for negative muon capture is then determined over the depth range defined below and for the altitude of the sampling location. The displayed depth range is for rock with a conservative high density of 2.7g/cm^3, so in most cases the fitted depth will be deeper than indicated. The graphs below show the quality of each fit to the Heisinger equations over the specified mass-depth. A default depth range is set at 20 m. At a minimum, the depth range over which the muon terms are fit should be equal to the depth of your deepest sample plus the maximum net erosion of the surface.

depth of muon fit:

(m)

muon stoopping rate (from Balco 2007 Matlab code):

effective atmospheric attenuation length for muons (from Balco 2007 Matlab code):

muon table from Groom et al. 2001

momentum:

attenuation lengths:

conversion of elevation to atmospheric pressure:

atmospheric depth:

vertical flux at SLHL:

stopping rate of vertical muons at site:

flux of vertical muons at site:

include constant flux >200,000:

total flux of muons at site (muons/cm2/a):

total stopping rate of muons at site:

negative muons (negmuons/g/a):

production rates:

fast muons (atoms/g/a) as a function of mass depth (z, g/cm2) and elevation (elev, m):

negative muon capture (atoms/g/a) as a function of mass depth (z, g/cm2) and elevation (elev, m):

two exponential term best fit to fast muons over specified mass depth and site elevation:

three exponential term best fit to negative muons over specified mass depth and site elevation:

total muon prodution rate from fitted curve and fractions for each exponential term:

summed muon attenuation lengths from fitted curve and fractions for each exponential term:

sample coefficients

Mean relative error (%) of fit over depth range:

Production rates and scaling:

**scaling scheme:**

You may chouse production rate scaling following Stone 2000 and enter SLHL production rates or skip Stone's scaling and enter your local production rates

How would you like to treat error in the production rates?

To treat as constant, enter the same high and low value, and 0 for the error; to treat as stochastic between high and low end members, enter the high and low values, and 0 for the error; to treat as normally distributed about a mean value, enter the mean value in both the high and low fields, and your estimate for the relative error. Muonic production rate and production rate of neutrons from U and Th decay can be treated as normally distributed about a mean value only, enter a normally distributed percent error in respective window.

spallogenic production rates:

**Ca**

**K**

**Ti**

**Fe**

**low**

(atoms g-1 a-1)

**high**

(atoms g-1 a-1)

**1****s** **error**

(atoms g-1 a-1)

production rate of epithermal neutrons from fast neutrons in the atmosphere at the land/atmosphere interface:

**low**

(atoms g-1 a-1)

**high**

(atoms g-1 a-1)

**1****s** **error**

(atoms g-1 a-1)

muonic production rate error:

**1****s** **error**

(%)

production rate error of neutrons from spontaneous fission of 238U and (a,n) reactions from U and Th decay series:

**1****s** **error**

(%)

Stone 2000:

a

b

c

d

e

m

Calculated production rates at surface (including shielding and cover):

spallogenic production rates:

**Ca**

**K**

**Ti**

**Fe**

**low**

(atoms g-1 a-1)

**high**

(atoms g-1 a-1)

**1****s** **error**

(atoms g-1 a-1)

production rate of epithermal neutrons from fast neutrons in the atmosphere at the landratmosphere interface:

**low**

(atoms g-1 a-1)

**high**

(atoms g-1 a-1)

**1****s** **error**

(atoms g-1 a-1)

attenuation length for neutrons:

fast neutrons

epithermal neutrons

thermal neutrons

the recommended attenuation length for your latitude is:

(g cm-2)

**low**

(g cm-2)

**high**

(g cm-2)

**1****s** **error**

(g cm-2)

Uncertainty in half life?

Half life is normally distributed about the 1s error specified (enter 0 to not include this error).

**1****s** **error**

(%)

The power coefficient of exponentially decreasing inheritance with depth C=Csurf\*exp(-(z/Λinh)).

(g cm-2)

neutrons produced by muons

spallogenic neutrons

neutrons from U and Th

36Cl production by neutron capture on 35Cl

тепловые нейтроны из термализованных быстрых по Гейзенгеру

Monte Carlo Simulator:

**parameter values for simulation:**

In the fields below, enter boundary conditions for the parameters you wish to stochastically simulate. To specify a known value for a parameter, enter the expected value in both the "low" and "high" fields; for this option you will have to enter 1s relative error in the expected value as well. You may constrain the simulation by erosion rate, as well as erosion.

age

net erosion

erosion rate

inheritance

**low**

(a)

(cm)

(cm ka-1)

(atoms g-1)

**high**

(a)

(cm)

(cm ka-1)

(atoms g-1)

**1****s** **error**

(a)

(cm ka-1)

(atoms g-1)

**random permutations:**

The simulation works by creating profiles from values sampled with the desired distribution from each of the above parameters. The total number of profiles needed to get a good grasp of your solution space depends on how well you can constrain each of the parameters. Thus, it is more useful to specify a population of profiles that fit within a certain degree of confidence for your data, than to specify a number of total random profiles to create. This simulation will generate a profile, generate a reduced chi-squared value from that profile, and determine if that value is as good or better than the value generated from the data in your profile at the confidence window you specify; it will then continue until (n) profiles pass this chi-squared test. The total number of profiles needed to collect (n) "good" profiles is also displayed (m).

**desired confidence (****s****):**

**define chi-squared:**

Guess value:

Solve Block:

**Max chi-squared value that will be collected\*:**

\*The simulation will only look for solutions with a lower reduced chi-squared value than that shown above. If your profile data is significantly scattered, then you may not be able to obtain low enough chi-squared values for certain confidence levels. In this case, it will be necessary to run the simulation at a higher cutoff for the chi-squared statistic. Although counter-intuitive, increasing your desired confidence level accomplishes this. This is easier to think of in terms of fitting curves to data points with error bars. A dataset may not allow any theoretical profile to fit the 1s (68% confidence) data error, but allow profiles to fit the larger 2s (95.4% confidence) error. Incidentally, a higher confidence results in a faster simulation as it is easier to find possible solutions within the larger error window.

**number of profiles within specified confidence:**

note: the best way to use this simulator is to start with low (n) values and do quick test simulations to tune the model to your data before running a very long high (n) value simulation. This allows you to do three things: 1) you can check the graphs below to see if the ranges you chose for your simulated parameters agree with the span of possible solutions for that parameter and then increase or decrease those ranges accordingly, 2) you can get an estimate for how long your simulation will take at higher (n) values (simulation time is linear), and 3) in the unfortunate circumstance of highly scattered data, you can run a quick test to see if it is even possible to find solutions better than your chi-squared cutoff (if it takes the program more than minute to find just 1 solution, then it probably will not be very useful for you to continue at that level of confidence). An (n) value of at least 100,000 is recommended for any final estimate of a given parameter.

Histograms:

Statistics:

Chi-squared plots:

Best fit profile:

Solution Space:

Age vs erosion rate:

Results:

**total number of simulated profiles:**

**Mode, mean, and median values for age, inheritance, and erosion rate:**

mean values:

median values:

modal values:

lowest c^2 value:

maximum value:

minumum value:

The range in age, inheritance, and erosion values shown in these plots represent the range over your specified confidence.

**parameters yielding best fit (lowest** **c****^2 value):**

c^2 value:

age:

erosion rate:

inheritance:

neutron atten. length:

spallogenic production rate:

muogenic production rate:

If you entered piecewise density data, the displayed values represent

mean cumulative bulk densities at the depth of each sample

density:

Note: the plot to the left may take a few minutes to generate if you have entered piecewise density values
