# Supplementary figures and images for: Fast long-term denudation rate of steep alpine headwalls inferred from cosmogenic 36Cl depth profiles (part 2 of 2)
Source: Sci Rep. 2019 Jul 30;9:11023. doi: 10.1038/s41598-019-46969-0 (PMC6667707; doi:10.1038/s41598-019-46969-0)

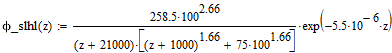

Supplement: Supplementary file 2 — Supplementary Dataset [file 41598_2019_46969_MOESM2_ESM.zip › S6 Monte Carlo input & results/EM02_mod_Rev3_20_full_images/IMG0138_401433593.PNG]

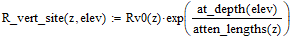

Supplement: Supplementary file 2 — Supplementary Dataset [file 41598_2019_46969_MOESM2_ESM.zip › S6 Monte Carlo input & results/EM02_mod_Rev3_20_full_images/IMG0139_401433593.PNG]

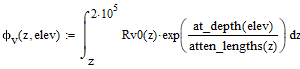

Supplement: Supplementary file 2 — Supplementary Dataset [file 41598_2019_46969_MOESM2_ESM.zip › S6 Monte Carlo input & results/EM02_mod_Rev3_20_full_images/IMG0140_401433593.PNG]

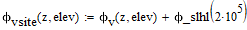

Supplement: Supplementary file 2 — Supplementary Dataset [file 41598_2019_46969_MOESM2_ESM.zip › S6 Monte Carlo input & results/EM02_mod_Rev3_20_full_images/IMG0141_401433593.PNG]

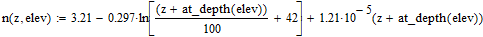

Supplement: Supplementary file 2 — Supplementary Dataset [file 41598_2019_46969_MOESM2_ESM.zip › S6 Monte Carlo input & results/EM02_mod_Rev3_20_full_images/IMG0142_401433593.PNG]

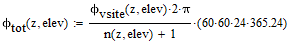

Supplement: Supplementary file 2 — Supplementary Dataset [file 41598_2019_46969_MOESM2_ESM.zip › S6 Monte Carlo input & results/EM02_mod_Rev3_20_full_images/IMG0143_401433593.PNG]

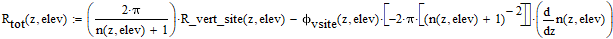

Supplement: Supplementary file 2 — Supplementary Dataset [file 41598_2019_46969_MOESM2_ESM.zip › S6 Monte Carlo input & results/EM02_mod_Rev3_20_full_images/IMG0144_401433609.PNG]

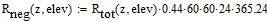

Supplement: Supplementary file 2 — Supplementary Dataset [file 41598_2019_46969_MOESM2_ESM.zip › S6 Monte Carlo input & results/EM02_mod_Rev3_20_full_images/IMG0145_401433609.PNG]

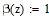

Supplement: Supplementary file 2 — Supplementary Dataset [file 41598_2019_46969_MOESM2_ESM.zip › S6 Monte Carlo input & results/EM02_mod_Rev3_20_full_images/IMG0146_401433609.PNG]

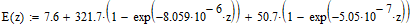

Supplement: Supplementary file 2 — Supplementary Dataset [file 41598_2019_46969_MOESM2_ESM.zip › S6 Monte Carlo input & results/EM02_mod_Rev3_20_full_images/IMG0147_401433609.PNG]

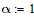

Supplement: Supplementary file 2 — Supplementary Dataset [file 41598_2019_46969_MOESM2_ESM.zip › S6 Monte Carlo input & results/EM02_mod_Rev3_20_full_images/IMG0148_401433609.PNG]

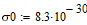

Supplement: Supplementary file 2 — Supplementary Dataset [file 41598_2019_46969_MOESM2_ESM.zip › S6 Monte Carlo input & results/EM02_mod_Rev3_20_full_images/IMG0149_401433609.PNG]

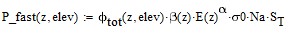

Supplement: Supplementary file 2 — Supplementary Dataset [file 41598_2019_46969_MOESM2_ESM.zip › S6 Monte Carlo input & results/EM02_mod_Rev3_20_full_images/IMG0150_401433609.PNG]

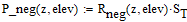

Supplement: Supplementary file 2 — Supplementary Dataset [file 41598_2019_46969_MOESM2_ESM.zip › S6 Monte Carlo input & results/EM02_mod_Rev3_20_full_images/IMG0151_401433609.PNG]

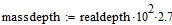

Supplement: Supplementary file 2 — Supplementary Dataset [file 41598_2019_46969_MOESM2_ESM.zip › S6 Monte Carlo input & results/EM02_mod_Rev3_20_full_images/IMG0152_401433625.PNG]

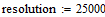

Supplement: Supplementary file 2 — Supplementary Dataset [file 41598_2019_46969_MOESM2_ESM.zip › S6 Monte Carlo input & results/EM02_mod_Rev3_20_full_images/IMG0153_401433625.PNG]

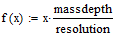

Supplement: Supplementary file 2 — Supplementary Dataset [file 41598_2019_46969_MOESM2_ESM.zip › S6 Monte Carlo input & results/EM02_mod_Rev3_20_full_images/IMG0154_401433625.PNG]

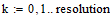

Supplement: Supplementary file 2 — Supplementary Dataset [file 41598_2019_46969_MOESM2_ESM.zip › S6 Monte Carlo input & results/EM02_mod_Rev3_20_full_images/IMG0155_401433625.PNG]

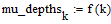

Supplement: Supplementary file 2 — Supplementary Dataset [file 41598_2019_46969_MOESM2_ESM.zip › S6 Monte Carlo input & results/EM02_mod_Rev3_20_full_images/IMG0156_401433625.PNG]

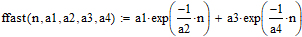

Supplement: Supplementary file 2 — Supplementary Dataset [file 41598_2019_46969_MOESM2_ESM.zip › S6 Monte Carlo input & results/EM02_mod_Rev3_20_full_images/IMG0157_401433625.PNG]

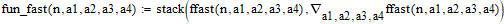

Supplement: Supplementary file 2 — Supplementary Dataset [file 41598_2019_46969_MOESM2_ESM.zip › S6 Monte Carlo input & results/EM02_mod_Rev3_20_full_images/IMG0158_401433625.PNG]

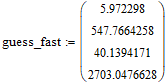

Supplement: Supplementary file 2 — Supplementary Dataset [file 41598_2019_46969_MOESM2_ESM.zip › S6 Monte Carlo input & results/EM02_mod_Rev3_20_full_images/IMG0159_401433625.PNG]

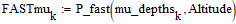

Supplement: Supplementary file 2 — Supplementary Dataset [file 41598_2019_46969_MOESM2_ESM.zip › S6 Monte Carlo input & results/EM02_mod_Rev3_20_full_images/IMG0160_401433640.PNG]

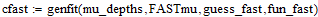

Supplement: Supplementary file 2 — Supplementary Dataset [file 41598_2019_46969_MOESM2_ESM.zip › S6 Monte Carlo input & results/EM02_mod_Rev3_20_full_images/IMG0161_401433640.PNG]

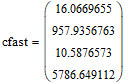

Supplement: Supplementary file 2 — Supplementary Dataset [file 41598_2019_46969_MOESM2_ESM.zip › S6 Monte Carlo input & results/EM02_mod_Rev3_20_full_images/IMG0162_401433640.PNG]

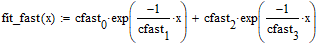

Supplement: Supplementary file 2 — Supplementary Dataset [file 41598_2019_46969_MOESM2_ESM.zip › S6 Monte Carlo input & results/EM02_mod_Rev3_20_full_images/IMG0163_401433640.PNG]

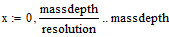

Supplement: Supplementary file 2 — Supplementary Dataset [file 41598_2019_46969_MOESM2_ESM.zip › S6 Monte Carlo input & results/EM02_mod_Rev3_20_full_images/IMG0164_401433640.PNG]

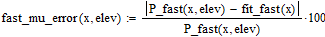

Supplement: Supplementary file 2 — Supplementary Dataset [file 41598_2019_46969_MOESM2_ESM.zip › S6 Monte Carlo input & results/EM02_mod_Rev3_20_full_images/IMG0165_401433640.PNG]

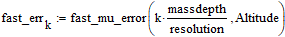

Supplement: Supplementary file 2 — Supplementary Dataset [file 41598_2019_46969_MOESM2_ESM.zip › S6 Monte Carlo input & results/EM02_mod_Rev3_20_full_images/IMG0166_401433640.PNG]

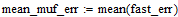

Supplement: Supplementary file 2 — Supplementary Dataset [file 41598_2019_46969_MOESM2_ESM.zip › S6 Monte Carlo input & results/EM02_mod_Rev3_20_full_images/IMG0167_401433640.PNG]

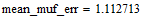

Supplement: Supplementary file 2 — Supplementary Dataset [file 41598_2019_46969_MOESM2_ESM.zip › S6 Monte Carlo input & results/EM02_mod_Rev3_20_full_images/IMG0168_401433640.PNG]

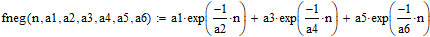

Supplement: Supplementary file 2 — Supplementary Dataset [file 41598_2019_46969_MOESM2_ESM.zip › S6 Monte Carlo input & results/EM02_mod_Rev3_20_full_images/IMG0169_401433640.PNG]

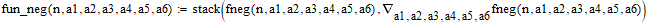

Supplement: Supplementary file 2 — Supplementary Dataset [file 41598_2019_46969_MOESM2_ESM.zip › S6 Monte Carlo input & results/EM02_mod_Rev3_20_full_images/IMG0170_401433656.PNG]

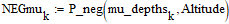

Supplement: Supplementary file 2 — Supplementary Dataset [file 41598_2019_46969_MOESM2_ESM.zip › S6 Monte Carlo input & results/EM02_mod_Rev3_20_full_images/IMG0171_401433656.PNG]

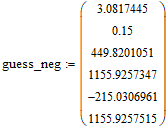

Supplement: Supplementary file 2 — Supplementary Dataset [file 41598_2019_46969_MOESM2_ESM.zip › S6 Monte Carlo input & results/EM02_mod_Rev3_20_full_images/IMG0172_401433656.PNG]

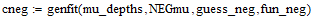

Supplement: Supplementary file 2 — Supplementary Dataset [file 41598_2019_46969_MOESM2_ESM.zip › S6 Monte Carlo input & results/EM02_mod_Rev3_20_full_images/IMG0173_401433656.PNG]

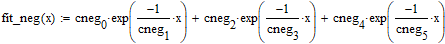

Supplement: Supplementary file 2 — Supplementary Dataset [file 41598_2019_46969_MOESM2_ESM.zip › S6 Monte Carlo input & results/EM02_mod_Rev3_20_full_images/IMG0174_401433656.PNG]

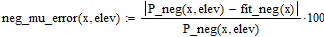

Supplement: Supplementary file 2 — Supplementary Dataset [file 41598_2019_46969_MOESM2_ESM.zip › S6 Monte Carlo input & results/EM02_mod_Rev3_20_full_images/IMG0175_401433656.PNG]

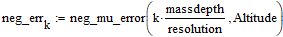

Supplement: Supplementary file 2 — Supplementary Dataset [file 41598_2019_46969_MOESM2_ESM.zip › S6 Monte Carlo input & results/EM02_mod_Rev3_20_full_images/IMG0176_401433656.PNG]

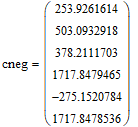

Supplement: Supplementary file 2 — Supplementary Dataset [file 41598_2019_46969_MOESM2_ESM.zip › S6 Monte Carlo input & results/EM02_mod_Rev3_20_full_images/IMG0177_401433656.PNG]

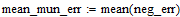

Supplement: Supplementary file 2 — Supplementary Dataset [file 41598_2019_46969_MOESM2_ESM.zip › S6 Monte Carlo input & results/EM02_mod_Rev3_20_full_images/IMG0178_401433656.PNG]

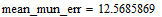

Supplement: Supplementary file 2 — Supplementary Dataset [file 41598_2019_46969_MOESM2_ESM.zip › S6 Monte Carlo input & results/EM02_mod_Rev3_20_full_images/IMG0179_401433671.PNG]

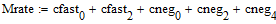

Supplement: Supplementary file 2 — Supplementary Dataset [file 41598_2019_46969_MOESM2_ESM.zip › S6 Monte Carlo input & results/EM02_mod_Rev3_20_full_images/IMG0180_401433671.PNG]

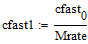

Supplement: Supplementary file 2 — Supplementary Dataset [file 41598_2019_46969_MOESM2_ESM.zip › S6 Monte Carlo input & results/EM02_mod_Rev3_20_full_images/IMG0181_401433671.PNG]

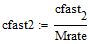

Supplement: Supplementary file 2 — Supplementary Dataset [file 41598_2019_46969_MOESM2_ESM.zip › S6 Monte Carlo input & results/EM02_mod_Rev3_20_full_images/IMG0182_401433671.PNG]

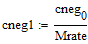

Supplement: Supplementary file 2 — Supplementary Dataset [file 41598_2019_46969_MOESM2_ESM.zip › S6 Monte Carlo input & results/EM02_mod_Rev3_20_full_images/IMG0183_401433671.PNG]

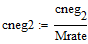

Supplement: Supplementary file 2 — Supplementary Dataset [file 41598_2019_46969_MOESM2_ESM.zip › S6 Monte Carlo input & results/EM02_mod_Rev3_20_full_images/IMG0184_401433671.PNG]

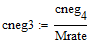

Supplement: Supplementary file 2 — Supplementary Dataset [file 41598_2019_46969_MOESM2_ESM.zip › S6 Monte Carlo input & results/EM02_mod_Rev3_20_full_images/IMG0185_401433671.PNG]

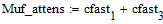

Supplement: Supplementary file 2 — Supplementary Dataset [file 41598_2019_46969_MOESM2_ESM.zip › S6 Monte Carlo input & results/EM02_mod_Rev3_20_full_images/IMG0186_401433671.PNG]

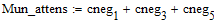

Supplement: Supplementary file 2 — Supplementary Dataset [file 41598_2019_46969_MOESM2_ESM.zip › S6 Monte Carlo input & results/EM02_mod_Rev3_20_full_images/IMG0187_401433671.PNG]

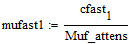

Supplement: Supplementary file 2 — Supplementary Dataset [file 41598_2019_46969_MOESM2_ESM.zip › S6 Monte Carlo input & results/EM02_mod_Rev3_20_full_images/IMG0188_401433687.PNG]

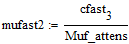

Supplement: Supplementary file 2 — Supplementary Dataset [file 41598_2019_46969_MOESM2_ESM.zip › S6 Monte Carlo input & results/EM02_mod_Rev3_20_full_images/IMG0189_401433687.PNG]

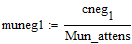

Supplement: Supplementary file 2 — Supplementary Dataset [file 41598_2019_46969_MOESM2_ESM.zip › S6 Monte Carlo input & results/EM02_mod_Rev3_20_full_images/IMG0190_401433687.PNG]

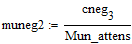

Supplement: Supplementary file 2 — Supplementary Dataset [file 41598_2019_46969_MOESM2_ESM.zip › S6 Monte Carlo input & results/EM02_mod_Rev3_20_full_images/IMG0191_401433687.PNG]

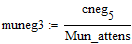

Supplement: Supplementary file 2 — Supplementary Dataset [file 41598_2019_46969_MOESM2_ESM.zip › S6 Monte Carlo input & results/EM02_mod_Rev3_20_full_images/IMG0192_401433687.PNG]

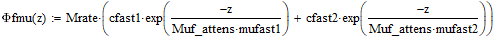

Supplement: Supplementary file 2 — Supplementary Dataset [file 41598_2019_46969_MOESM2_ESM.zip › S6 Monte Carlo input & results/EM02_mod_Rev3_20_full_images/IMG0194_401433687.PNG]

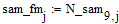

Supplement: Supplementary file 2 — Supplementary Dataset [file 41598_2019_46969_MOESM2_ESM.zip › S6 Monte Carlo input & results/EM02_mod_Rev3_20_full_images/IMG0195_401433687.PNG]

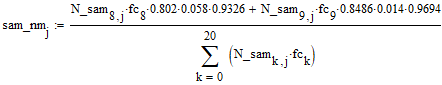

Supplement: Supplementary file 2 — Supplementary Dataset [file 41598_2019_46969_MOESM2_ESM.zip › S6 Monte Carlo input & results/EM02_mod_Rev3_20_full_images/IMG0196_401433687.PNG]

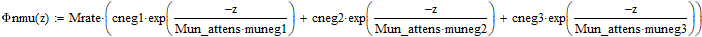

Supplement: Supplementary file 2 — Supplementary Dataset [file 41598_2019_46969_MOESM2_ESM.zip › S6 Monte Carlo input & results/EM02_mod_Rev3_20_full_images/IMG0197_401433687.PNG]

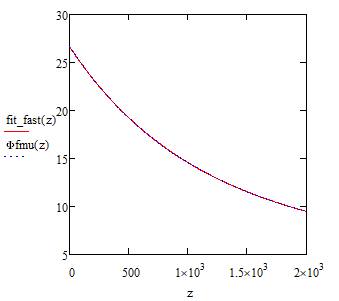

Supplement: Supplementary file 2 — Supplementary Dataset [file 41598_2019_46969_MOESM2_ESM.zip › S6 Monte Carlo input & results/EM02_mod_Rev3_20_full_images/IMG0199_401433703.PNG]

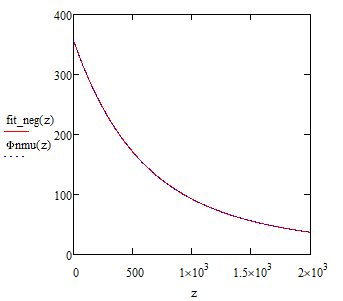

Supplement: Supplementary file 2 — Supplementary Dataset [file 41598_2019_46969_MOESM2_ESM.zip › S6 Monte Carlo input & results/EM02_mod_Rev3_20_full_images/IMG0201_401433703.PNG]

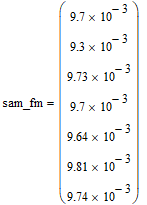

Supplement: Supplementary file 2 — Supplementary Dataset [file 41598_2019_46969_MOESM2_ESM.zip › S6 Monte Carlo input & results/EM02_mod_Rev3_20_full_images/IMG0202_401433703.PNG]

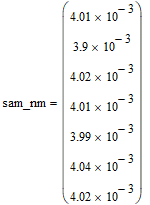

Supplement: Supplementary file 2 — Supplementary Dataset [file 41598_2019_46969_MOESM2_ESM.zip › S6 Monte Carlo input & results/EM02_mod_Rev3_20_full_images/IMG0203_401433703.PNG]

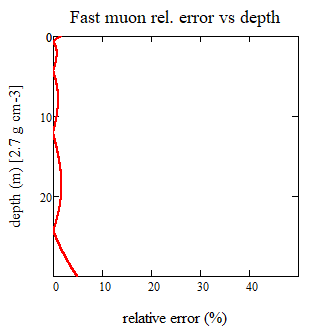

Supplement: Supplementary file 2 — Supplementary Dataset [file 41598_2019_46969_MOESM2_ESM.zip › S6 Monte Carlo input & results/EM02_mod_Rev3_20_full_images/IMG0208_401433703.PNG]

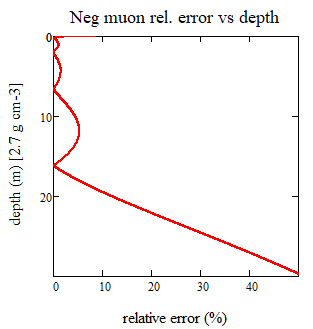

Supplement: Supplementary file 2 — Supplementary Dataset [file 41598_2019_46969_MOESM2_ESM.zip › S6 Monte Carlo input & results/EM02_mod_Rev3_20_full_images/IMG0210_401433703.PNG]

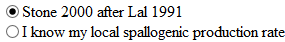

Supplement: Supplementary file 2 — Supplementary Dataset [file 41598_2019_46969_MOESM2_ESM.zip › S6 Monte Carlo input & results/EM02_mod_Rev3_20_full_images/IMG0212_401433703.PNG]

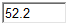

Supplement: Supplementary file 2 — Supplementary Dataset [file 41598_2019_46969_MOESM2_ESM.zip › S6 Monte Carlo input & results/EM02_mod_Rev3_20_full_images/IMG0214_401433718.PNG]

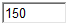

Supplement: Supplementary file 2 — Supplementary Dataset [file 41598_2019_46969_MOESM2_ESM.zip › S6 Monte Carlo input & results/EM02_mod_Rev3_20_full_images/IMG0216_401433718.PNG]

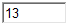

Supplement: Supplementary file 2 — Supplementary Dataset [file 41598_2019_46969_MOESM2_ESM.zip › S6 Monte Carlo input & results/EM02_mod_Rev3_20_full_images/IMG0218_401433718.PNG]

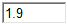

Supplement: Supplementary file 2 — Supplementary Dataset [file 41598_2019_46969_MOESM2_ESM.zip › S6 Monte Carlo input & results/EM02_mod_Rev3_20_full_images/IMG0220_401433718.PNG]

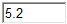

Supplement: Supplementary file 2 — Supplementary Dataset [file 41598_2019_46969_MOESM2_ESM.zip › S6 Monte Carlo input & results/EM02_mod_Rev3_20_full_images/IMG0230_401433734.PNG]

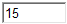

Supplement: Supplementary file 2 — Supplementary Dataset [file 41598_2019_46969_MOESM2_ESM.zip › S6 Monte Carlo input & results/EM02_mod_Rev3_20_full_images/IMG0232_401433734.PNG]

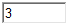

Supplement: Supplementary file 2 — Supplementary Dataset [file 41598_2019_46969_MOESM2_ESM.zip › S6 Monte Carlo input & results/EM02_mod_Rev3_20_full_images/IMG0234_401433734.PNG]

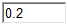

Supplement: Supplementary file 2 — Supplementary Dataset [file 41598_2019_46969_MOESM2_ESM.zip › S6 Monte Carlo input & results/EM02_mod_Rev3_20_full_images/IMG0236_401433734.PNG]

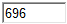

Supplement: Supplementary file 2 — Supplementary Dataset [file 41598_2019_46969_MOESM2_ESM.zip › S6 Monte Carlo input & results/EM02_mod_Rev3_20_full_images/IMG0238_401433734.PNG]

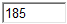

Supplement: Supplementary file 2 — Supplementary Dataset [file 41598_2019_46969_MOESM2_ESM.zip › S6 Monte Carlo input & results/EM02_mod_Rev3_20_full_images/IMG0242_401433734.PNG]

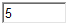

Supplement: Supplementary file 2 — Supplementary Dataset [file 41598_2019_46969_MOESM2_ESM.zip › S6 Monte Carlo input & results/EM02_mod_Rev3_20_full_images/IMG0244_401433734.PNG]

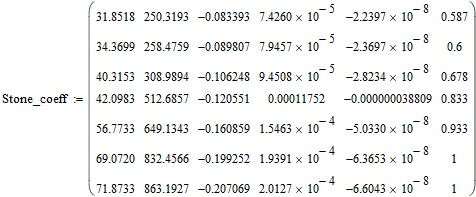

Supplement: Supplementary file 2 — Supplementary Dataset [file 41598_2019_46969_MOESM2_ESM.zip › S6 Monte Carlo input & results/EM02_mod_Rev3_20_full_images/IMG0247_401433750.PNG]

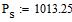

Supplement: Supplementary file 2 — Supplementary Dataset [file 41598_2019_46969_MOESM2_ESM.zip › S6 Monte Carlo input & results/EM02_mod_Rev3_20_full_images/IMG0248_401433750.PNG]
