# Supplementary figures and images for: Fast long-term denudation rate of steep alpine headwalls inferred from cosmogenic 36Cl depth profiles (part 1 of 2)
Source: Sci Rep. 2019 Jul 30;9:11023. doi: 10.1038/s41598-019-46969-0 (PMC6667707; doi:10.1038/s41598-019-46969-0)

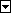

Supplement: Supplementary file 2 — Supplementary Dataset [file 41598_2019_46969_MOESM2_ESM.zip › S6 Monte Carlo input & results/EM02_mod_Rev3_20_full_images/IMG0000_401433437.PNG]

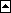

Supplement: Supplementary file 2 — Supplementary Dataset [file 41598_2019_46969_MOESM2_ESM.zip › S6 Monte Carlo input & results/EM02_mod_Rev3_20_full_images/IMG0000_401433515.PNG]

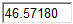

Supplement: Supplementary file 2 — Supplementary Dataset [file 41598_2019_46969_MOESM2_ESM.zip › S6 Monte Carlo input & results/EM02_mod_Rev3_20_full_images/IMG0004_401433406.PNG]

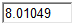

Supplement: Supplementary file 2 — Supplementary Dataset [file 41598_2019_46969_MOESM2_ESM.zip › S6 Monte Carlo input & results/EM02_mod_Rev3_20_full_images/IMG0006_401433406.PNG]

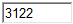

Supplement: Supplementary file 2 — Supplementary Dataset [file 41598_2019_46969_MOESM2_ESM.zip › S6 Monte Carlo input & results/EM02_mod_Rev3_20_full_images/IMG0008_401433406.PNG]

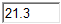

Supplement: Supplementary file 2 — Supplementary Dataset [file 41598_2019_46969_MOESM2_ESM.zip › S6 Monte Carlo input & results/EM02_mod_Rev3_20_full_images/IMG0010_401433421.PNG]

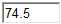

Supplement: Supplementary file 2 — Supplementary Dataset [file 41598_2019_46969_MOESM2_ESM.zip › S6 Monte Carlo input & results/EM02_mod_Rev3_20_full_images/IMG0012_401433421.PNG]

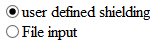

Supplement: Supplementary file 2 — Supplementary Dataset [file 41598_2019_46969_MOESM2_ESM.zip › S6 Monte Carlo input & results/EM02_mod_Rev3_20_full_images/IMG0014_401433421.PNG]

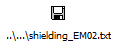

Supplement: Supplementary file 2 — Supplementary Dataset [file 41598_2019_46969_MOESM2_ESM.zip › S6 Monte Carlo input & results/EM02_mod_Rev3_20_full_images/IMG0016_401433421.PNG]

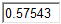

Supplement: Supplementary file 2 — Supplementary Dataset [file 41598_2019_46969_MOESM2_ESM.zip › S6 Monte Carlo input & results/EM02_mod_Rev3_20_full_images/IMG0018_401433421.PNG]

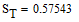

Supplement: Supplementary file 2 — Supplementary Dataset [file 41598_2019_46969_MOESM2_ESM.zip › S6 Monte Carlo input & results/EM02_mod_Rev3_20_full_images/IMG0020_401433421.PNG]

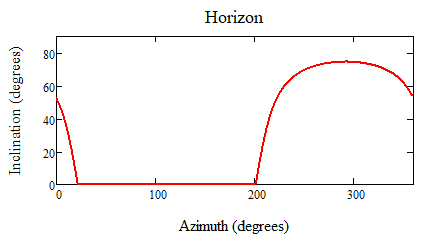

Supplement: Supplementary file 2 — Supplementary Dataset [file 41598_2019_46969_MOESM2_ESM.zip › S6 Monte Carlo input & results/EM02_mod_Rev3_20_full_images/IMG0022_401433421.PNG]

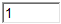

Supplement: Supplementary file 2 — Supplementary Dataset [file 41598_2019_46969_MOESM2_ESM.zip › S6 Monte Carlo input & results/EM02_mod_Rev3_20_full_images/IMG0024_401433421.PNG]

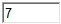

Supplement: Supplementary file 2 — Supplementary Dataset [file 41598_2019_46969_MOESM2_ESM.zip › S6 Monte Carlo input & results/EM02_mod_Rev3_20_full_images/IMG0026_401433421.PNG]

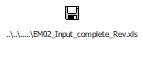

Supplement: Supplementary file 2 — Supplementary Dataset [file 41598_2019_46969_MOESM2_ESM.zip › S6 Monte Carlo input & results/EM02_mod_Rev3_20_full_images/IMG0028_401433421.PNG]

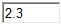

Supplement: Supplementary file 2 — Supplementary Dataset [file 41598_2019_46969_MOESM2_ESM.zip › S6 Monte Carlo input & results/EM02_mod_Rev3_20_full_images/IMG0030_401433421.PNG]

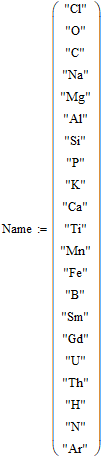

Supplement: Supplementary file 2 — Supplementary Dataset [file 41598_2019_46969_MOESM2_ESM.zip › S6 Monte Carlo input & results/EM02_mod_Rev3_20_full_images/IMG0031_401433437.PNG]

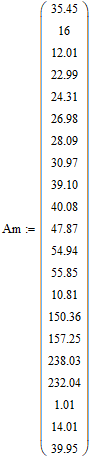

Supplement: Supplementary file 2 — Supplementary Dataset [file 41598_2019_46969_MOESM2_ESM.zip › S6 Monte Carlo input & results/EM02_mod_Rev3_20_full_images/IMG0032_401433437.PNG]

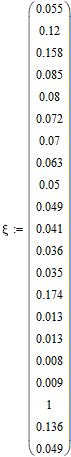

Supplement: Supplementary file 2 — Supplementary Dataset [file 41598_2019_46969_MOESM2_ESM.zip › S6 Monte Carlo input & results/EM02_mod_Rev3_20_full_images/IMG0033_401433437.PNG]

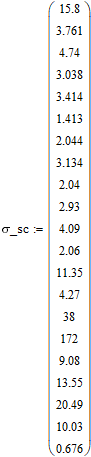

Supplement: Supplementary file 2 — Supplementary Dataset [file 41598_2019_46969_MOESM2_ESM.zip › S6 Monte Carlo input & results/EM02_mod_Rev3_20_full_images/IMG0034_401433437.PNG]

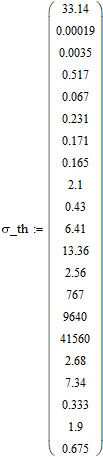

Supplement: Supplementary file 2 — Supplementary Dataset [file 41598_2019_46969_MOESM2_ESM.zip › S6 Monte Carlo input & results/EM02_mod_Rev3_20_full_images/IMG0035_401433437.PNG]

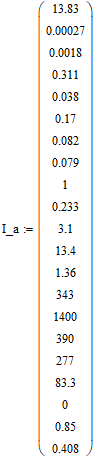

Supplement: Supplementary file 2 — Supplementary Dataset [file 41598_2019_46969_MOESM2_ESM.zip › S6 Monte Carlo input & results/EM02_mod_Rev3_20_full_images/IMG0036_401433437.PNG]

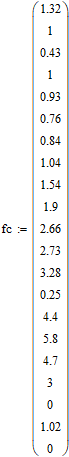

Supplement: Supplementary file 2 — Supplementary Dataset [file 41598_2019_46969_MOESM2_ESM.zip › S6 Monte Carlo input & results/EM02_mod_Rev3_20_full_images/IMG0038_401433437.PNG]

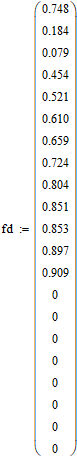

Supplement: Supplementary file 2 — Supplementary Dataset [file 41598_2019_46969_MOESM2_ESM.zip › S6 Monte Carlo input & results/EM02_mod_Rev3_20_full_images/IMG0039_401433437.PNG]

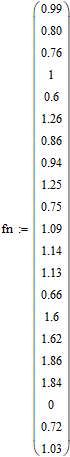

Supplement: Supplementary file 2 — Supplementary Dataset [file 41598_2019_46969_MOESM2_ESM.zip › S6 Monte Carlo input & results/EM02_mod_Rev3_20_full_images/IMG0040_401433453.PNG]

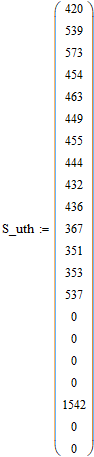

Supplement: Supplementary file 2 — Supplementary Dataset [file 41598_2019_46969_MOESM2_ESM.zip › S6 Monte Carlo input & results/EM02_mod_Rev3_20_full_images/IMG0041_401433453.PNG]

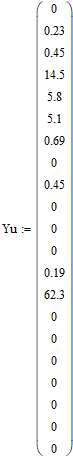

Supplement: Supplementary file 2 — Supplementary Dataset [file 41598_2019_46969_MOESM2_ESM.zip › S6 Monte Carlo input & results/EM02_mod_Rev3_20_full_images/IMG0042_401433453.PNG]

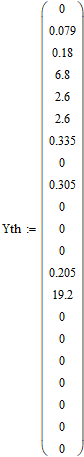

Supplement: Supplementary file 2 — Supplementary Dataset [file 41598_2019_46969_MOESM2_ESM.zip › S6 Monte Carlo input & results/EM02_mod_Rev3_20_full_images/IMG0043_401433453.PNG]

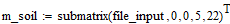

Supplement: Supplementary file 2 — Supplementary Dataset [file 41598_2019_46969_MOESM2_ESM.zip › S6 Monte Carlo input & results/EM02_mod_Rev3_20_full_images/IMG0044_401433453.PNG]

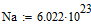

Supplement: Supplementary file 2 — Supplementary Dataset [file 41598_2019_46969_MOESM2_ESM.zip › S6 Monte Carlo input & results/EM02_mod_Rev3_20_full_images/IMG0045_401433453.PNG]

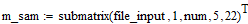

Supplement: Supplementary file 2 — Supplementary Dataset [file 41598_2019_46969_MOESM2_ESM.zip › S6 Monte Carlo input & results/EM02_mod_Rev3_20_full_images/IMG0046_401433453.PNG]

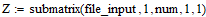

Supplement: Supplementary file 2 — Supplementary Dataset [file 41598_2019_46969_MOESM2_ESM.zip › S6 Monte Carlo input & results/EM02_mod_Rev3_20_full_images/IMG0047_401433453.PNG]

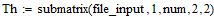

Supplement: Supplementary file 2 — Supplementary Dataset [file 41598_2019_46969_MOESM2_ESM.zip › S6 Monte Carlo input & results/EM02_mod_Rev3_20_full_images/IMG0048_401433453.PNG]

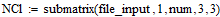

Supplement: Supplementary file 2 — Supplementary Dataset [file 41598_2019_46969_MOESM2_ESM.zip › S6 Monte Carlo input & results/EM02_mod_Rev3_20_full_images/IMG0049_401433453.PNG]

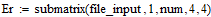

Supplement: Supplementary file 2 — Supplementary Dataset [file 41598_2019_46969_MOESM2_ESM.zip › S6 Monte Carlo input & results/EM02_mod_Rev3_20_full_images/IMG0050_401433468.PNG]

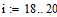

Supplement: Supplementary file 2 — Supplementary Dataset [file 41598_2019_46969_MOESM2_ESM.zip › S6 Monte Carlo input & results/EM02_mod_Rev3_20_full_images/IMG0051_401433468.PNG]

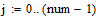

Supplement: Supplementary file 2 — Supplementary Dataset [file 41598_2019_46969_MOESM2_ESM.zip › S6 Monte Carlo input & results/EM02_mod_Rev3_20_full_images/IMG0052_401433468.PNG]

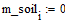

Supplement: Supplementary file 2 — Supplementary Dataset [file 41598_2019_46969_MOESM2_ESM.zip › S6 Monte Carlo input & results/EM02_mod_Rev3_20_full_images/IMG0053_401433468.PNG]

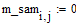

Supplement: Supplementary file 2 — Supplementary Dataset [file 41598_2019_46969_MOESM2_ESM.zip › S6 Monte Carlo input & results/EM02_mod_Rev3_20_full_images/IMG0054_401433468.PNG]

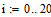

Supplement: Supplementary file 2 — Supplementary Dataset [file 41598_2019_46969_MOESM2_ESM.zip › S6 Monte Carlo input & results/EM02_mod_Rev3_20_full_images/IMG0055_401433468.PNG]

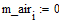

Supplement: Supplementary file 2 — Supplementary Dataset [file 41598_2019_46969_MOESM2_ESM.zip › S6 Monte Carlo input & results/EM02_mod_Rev3_20_full_images/IMG0056_401433468.PNG]

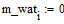

Supplement: Supplementary file 2 — Supplementary Dataset [file 41598_2019_46969_MOESM2_ESM.zip › S6 Monte Carlo input & results/EM02_mod_Rev3_20_full_images/IMG0057_401433468.PNG]

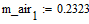

Supplement: Supplementary file 2 — Supplementary Dataset [file 41598_2019_46969_MOESM2_ESM.zip › S6 Monte Carlo input & results/EM02_mod_Rev3_20_full_images/IMG0058_401433484.PNG]

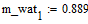

Supplement: Supplementary file 2 — Supplementary Dataset [file 41598_2019_46969_MOESM2_ESM.zip › S6 Monte Carlo input & results/EM02_mod_Rev3_20_full_images/IMG0059_401433484.PNG]

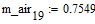

Supplement: Supplementary file 2 — Supplementary Dataset [file 41598_2019_46969_MOESM2_ESM.zip › S6 Monte Carlo input & results/EM02_mod_Rev3_20_full_images/IMG0060_401433484.PNG]

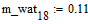

Supplement: Supplementary file 2 — Supplementary Dataset [file 41598_2019_46969_MOESM2_ESM.zip › S6 Monte Carlo input & results/EM02_mod_Rev3_20_full_images/IMG0061_401433484.PNG]

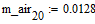

Supplement: Supplementary file 2 — Supplementary Dataset [file 41598_2019_46969_MOESM2_ESM.zip › S6 Monte Carlo input & results/EM02_mod_Rev3_20_full_images/IMG0062_401433484.PNG]

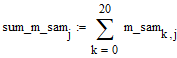

Supplement: Supplementary file 2 — Supplementary Dataset [file 41598_2019_46969_MOESM2_ESM.zip › S6 Monte Carlo input & results/EM02_mod_Rev3_20_full_images/IMG0063_401433484.PNG]

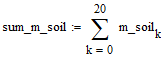

Supplement: Supplementary file 2 — Supplementary Dataset [file 41598_2019_46969_MOESM2_ESM.zip › S6 Monte Carlo input & results/EM02_mod_Rev3_20_full_images/IMG0064_401433484.PNG]

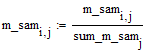

Supplement: Supplementary file 2 — Supplementary Dataset [file 41598_2019_46969_MOESM2_ESM.zip › S6 Monte Carlo input & results/EM02_mod_Rev3_20_full_images/IMG0065_401433500.PNG]

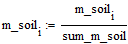

Supplement: Supplementary file 2 — Supplementary Dataset [file 41598_2019_46969_MOESM2_ESM.zip › S6 Monte Carlo input & results/EM02_mod_Rev3_20_full_images/IMG0066_401433500.PNG]

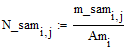

Supplement: Supplementary file 2 — Supplementary Dataset [file 41598_2019_46969_MOESM2_ESM.zip › S6 Monte Carlo input & results/EM02_mod_Rev3_20_full_images/IMG0067_401433500.PNG]

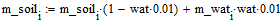

Supplement: Supplementary file 2 — Supplementary Dataset [file 41598_2019_46969_MOESM2_ESM.zip › S6 Monte Carlo input & results/EM02_mod_Rev3_20_full_images/IMG0068_401433500.PNG]

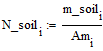

Supplement: Supplementary file 2 — Supplementary Dataset [file 41598_2019_46969_MOESM2_ESM.zip › S6 Monte Carlo input & results/EM02_mod_Rev3_20_full_images/IMG0071_401433500.PNG]

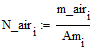

Supplement: Supplementary file 2 — Supplementary Dataset [file 41598_2019_46969_MOESM2_ESM.zip › S6 Monte Carlo input & results/EM02_mod_Rev3_20_full_images/IMG0072_401433500.PNG]

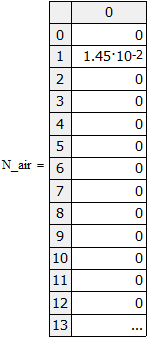

Supplement: Supplementary file 2 — Supplementary Dataset [file 41598_2019_46969_MOESM2_ESM.zip › S6 Monte Carlo input & results/EM02_mod_Rev3_20_full_images/IMG0074_401433515.PNG]

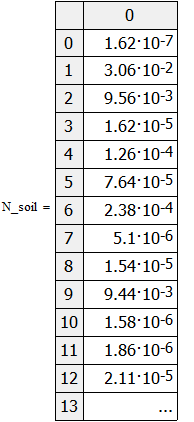

Supplement: Supplementary file 2 — Supplementary Dataset [file 41598_2019_46969_MOESM2_ESM.zip › S6 Monte Carlo input & results/EM02_mod_Rev3_20_full_images/IMG0076_401433515.PNG]

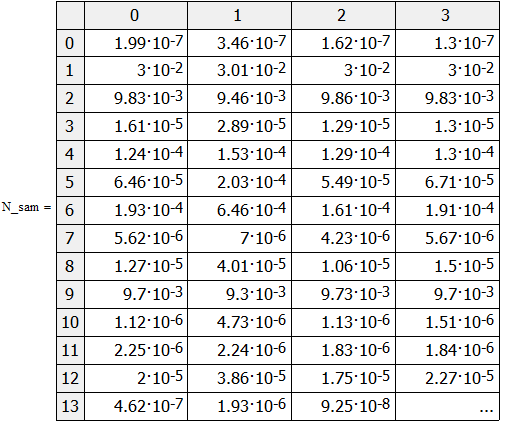

Supplement: Supplementary file 2 — Supplementary Dataset [file 41598_2019_46969_MOESM2_ESM.zip › S6 Monte Carlo input & results/EM02_mod_Rev3_20_full_images/IMG0078_401433515.PNG]

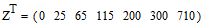

Supplement: Supplementary file 2 — Supplementary Dataset [file 41598_2019_46969_MOESM2_ESM.zip › S6 Monte Carlo input & results/EM02_mod_Rev3_20_full_images/IMG0080_401433515.PNG]

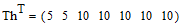

Supplement: Supplementary file 2 — Supplementary Dataset [file 41598_2019_46969_MOESM2_ESM.zip › S6 Monte Carlo input & results/EM02_mod_Rev3_20_full_images/IMG0081_401433515.PNG]

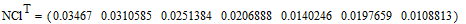

Supplement: Supplementary file 2 — Supplementary Dataset [file 41598_2019_46969_MOESM2_ESM.zip › S6 Monte Carlo input & results/EM02_mod_Rev3_20_full_images/IMG0082_401433515.PNG]

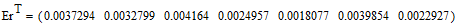

Supplement: Supplementary file 2 — Supplementary Dataset [file 41598_2019_46969_MOESM2_ESM.zip › S6 Monte Carlo input & results/EM02_mod_Rev3_20_full_images/IMG0083_401433515.PNG]

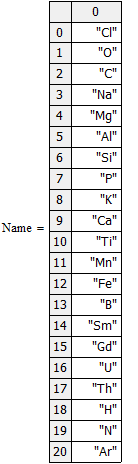

Supplement: Supplementary file 2 — Supplementary Dataset [file 41598_2019_46969_MOESM2_ESM.zip › S6 Monte Carlo input & results/EM02_mod_Rev3_20_full_images/IMG0085_401433515.PNG]

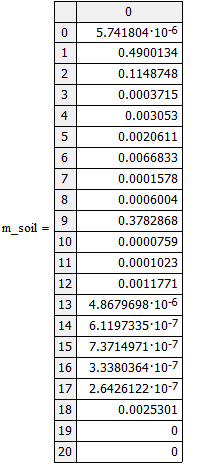

Supplement: Supplementary file 2 — Supplementary Dataset [file 41598_2019_46969_MOESM2_ESM.zip › S6 Monte Carlo input & results/EM02_mod_Rev3_20_full_images/IMG0087_401433515.PNG]

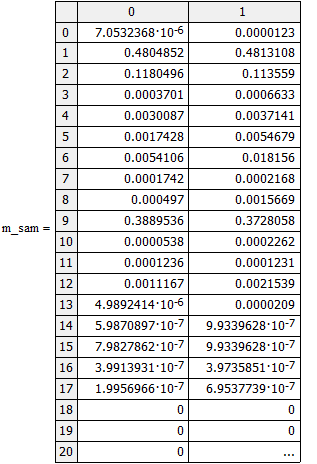

Supplement: Supplementary file 2 — Supplementary Dataset [file 41598_2019_46969_MOESM2_ESM.zip › S6 Monte Carlo input & results/EM02_mod_Rev3_20_full_images/IMG0089_401433531.PNG]

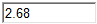

Supplement: Supplementary file 2 — Supplementary Dataset [file 41598_2019_46969_MOESM2_ESM.zip › S6 Monte Carlo input & results/EM02_mod_Rev3_20_full_images/IMG0091_401433531.PNG]

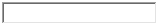

Supplement: Supplementary file 2 — Supplementary Dataset [file 41598_2019_46969_MOESM2_ESM.zip › S6 Monte Carlo input & results/EM02_mod_Rev3_20_full_images/IMG0093_401433531.PNG]

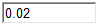

Supplement: Supplementary file 2 — Supplementary Dataset [file 41598_2019_46969_MOESM2_ESM.zip › S6 Monte Carlo input & results/EM02_mod_Rev3_20_full_images/IMG0099_401433531.PNG]

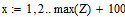

Supplement: Supplementary file 2 — Supplementary Dataset [file 41598_2019_46969_MOESM2_ESM.zip › S6 Monte Carlo input & results/EM02_mod_Rev3_20_full_images/IMG0102_401433531.PNG]

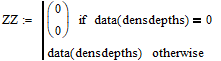

Supplement: Supplementary file 2 — Supplementary Dataset [file 41598_2019_46969_MOESM2_ESM.zip › S6 Monte Carlo input & results/EM02_mod_Rev3_20_full_images/IMG0103_401433531.PNG]

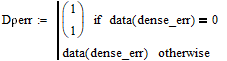

Supplement: Supplementary file 2 — Supplementary Dataset [file 41598_2019_46969_MOESM2_ESM.zip › S6 Monte Carlo input & results/EM02_mod_Rev3_20_full_images/IMG0104_401433531.PNG]

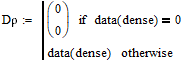

Supplement: Supplementary file 2 — Supplementary Dataset [file 41598_2019_46969_MOESM2_ESM.zip › S6 Monte Carlo input & results/EM02_mod_Rev3_20_full_images/IMG0105_401433546.PNG]

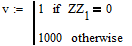

Supplement: Supplementary file 2 — Supplementary Dataset [file 41598_2019_46969_MOESM2_ESM.zip › S6 Monte Carlo input & results/EM02_mod_Rev3_20_full_images/IMG0106_401433546.PNG]

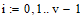

Supplement: Supplementary file 2 — Supplementary Dataset [file 41598_2019_46969_MOESM2_ESM.zip › S6 Monte Carlo input & results/EM02_mod_Rev3_20_full_images/IMG0107_401433546.PNG]

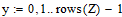

Supplement: Supplementary file 2 — Supplementary Dataset [file 41598_2019_46969_MOESM2_ESM.zip › S6 Monte Carlo input & results/EM02_mod_Rev3_20_full_images/IMG0108_401433546.PNG]

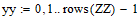

Supplement: Supplementary file 2 — Supplementary Dataset [file 41598_2019_46969_MOESM2_ESM.zip › S6 Monte Carlo input & results/EM02_mod_Rev3_20_full_images/IMG0109_401433546.PNG]

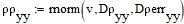

Supplement: Supplementary file 2 — Supplementary Dataset [file 41598_2019_46969_MOESM2_ESM.zip › S6 Monte Carlo input & results/EM02_mod_Rev3_20_full_images/IMG0110_401433546.PNG]

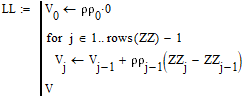

Supplement: Supplementary file 2 — Supplementary Dataset [file 41598_2019_46969_MOESM2_ESM.zip › S6 Monte Carlo input & results/EM02_mod_Rev3_20_full_images/IMG0111_401433546.PNG]

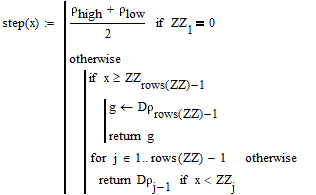

Supplement: Supplementary file 2 — Supplementary Dataset [file 41598_2019_46969_MOESM2_ESM.zip › S6 Monte Carlo input & results/EM02_mod_Rev3_20_full_images/IMG0112_401433562.PNG]

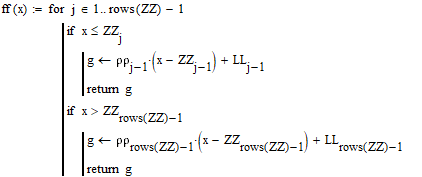

Supplement: Supplementary file 2 — Supplementary Dataset [file 41598_2019_46969_MOESM2_ESM.zip › S6 Monte Carlo input & results/EM02_mod_Rev3_20_full_images/IMG0113_401433562.PNG]

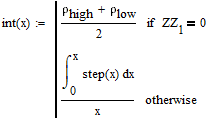

Supplement: Supplementary file 2 — Supplementary Dataset [file 41598_2019_46969_MOESM2_ESM.zip › S6 Monte Carlo input & results/EM02_mod_Rev3_20_full_images/IMG0114_401433562.PNG]

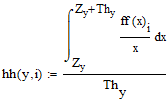

Supplement: Supplementary file 2 — Supplementary Dataset [file 41598_2019_46969_MOESM2_ESM.zip › S6 Monte Carlo input & results/EM02_mod_Rev3_20_full_images/IMG0115_401433562.PNG]

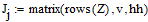

Supplement: Supplementary file 2 — Supplementary Dataset [file 41598_2019_46969_MOESM2_ESM.zip › S6 Monte Carlo input & results/EM02_mod_Rev3_20_full_images/IMG0116_401433562.PNG]

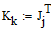

Supplement: Supplementary file 2 — Supplementary Dataset [file 41598_2019_46969_MOESM2_ESM.zip › S6 Monte Carlo input & results/EM02_mod_Rev3_20_full_images/IMG0117_401433562.PNG]

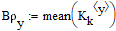

Supplement: Supplementary file 2 — Supplementary Dataset [file 41598_2019_46969_MOESM2_ESM.zip › S6 Monte Carlo input & results/EM02_mod_Rev3_20_full_images/IMG0118_401433562.PNG]

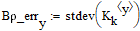

Supplement: Supplementary file 2 — Supplementary Dataset [file 41598_2019_46969_MOESM2_ESM.zip › S6 Monte Carlo input & results/EM02_mod_Rev3_20_full_images/IMG0119_401433562.PNG]

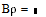

Supplement: Supplementary file 2 — Supplementary Dataset [file 41598_2019_46969_MOESM2_ESM.zip › S6 Monte Carlo input & results/EM02_mod_Rev3_20_full_images/IMG0120_401433562.PNG]

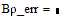

Supplement: Supplementary file 2 — Supplementary Dataset [file 41598_2019_46969_MOESM2_ESM.zip › S6 Monte Carlo input & results/EM02_mod_Rev3_20_full_images/IMG0121_401433562.PNG]

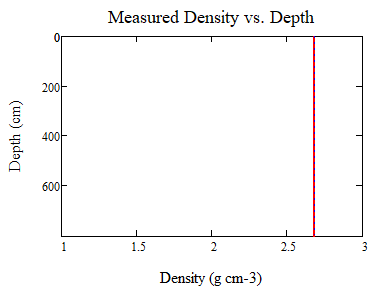

Supplement: Supplementary file 2 — Supplementary Dataset [file 41598_2019_46969_MOESM2_ESM.zip › S6 Monte Carlo input & results/EM02_mod_Rev3_20_full_images/IMG0124_401433578.PNG]

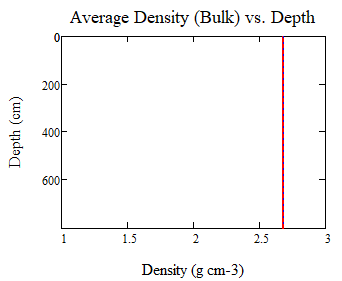

Supplement: Supplementary file 2 — Supplementary Dataset [file 41598_2019_46969_MOESM2_ESM.zip › S6 Monte Carlo input & results/EM02_mod_Rev3_20_full_images/IMG0126_401433578.PNG]

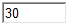

Supplement: Supplementary file 2 — Supplementary Dataset [file 41598_2019_46969_MOESM2_ESM.zip › S6 Monte Carlo input & results/EM02_mod_Rev3_20_full_images/IMG0128_401433578.PNG]

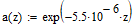

Supplement: Supplementary file 2 — Supplementary Dataset [file 41598_2019_46969_MOESM2_ESM.zip › S6 Monte Carlo input & results/EM02_mod_Rev3_20_full_images/IMG0129_401433578.PNG]

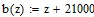

Supplement: Supplementary file 2 — Supplementary Dataset [file 41598_2019_46969_MOESM2_ESM.zip › S6 Monte Carlo input & results/EM02_mod_Rev3_20_full_images/IMG0130_401433578.PNG]

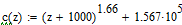

Supplement: Supplementary file 2 — Supplementary Dataset [file 41598_2019_46969_MOESM2_ESM.zip › S6 Monte Carlo input & results/EM02_mod_Rev3_20_full_images/IMG0131_401433578.PNG]

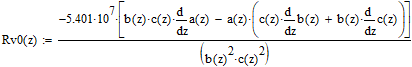

Supplement: Supplementary file 2 — Supplementary Dataset [file 41598_2019_46969_MOESM2_ESM.zip › S6 Monte Carlo input & results/EM02_mod_Rev3_20_full_images/IMG0132_401433578.PNG]

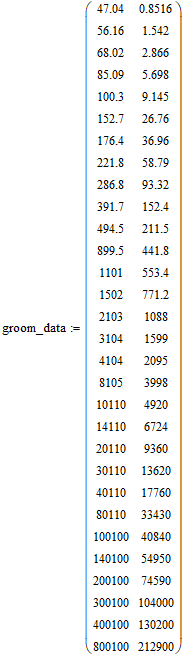

Supplement: Supplementary file 2 — Supplementary Dataset [file 41598_2019_46969_MOESM2_ESM.zip › S6 Monte Carlo input & results/EM02_mod_Rev3_20_full_images/IMG0133_401433578.PNG]

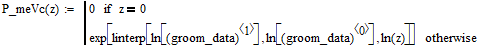

Supplement: Supplementary file 2 — Supplementary Dataset [file 41598_2019_46969_MOESM2_ESM.zip › S6 Monte Carlo input & results/EM02_mod_Rev3_20_full_images/IMG0134_401433578.PNG]

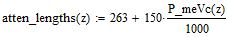

Supplement: Supplementary file 2 — Supplementary Dataset [file 41598_2019_46969_MOESM2_ESM.zip › S6 Monte Carlo input & results/EM02_mod_Rev3_20_full_images/IMG0135_401433578.PNG]

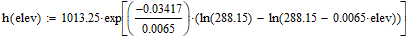

Supplement: Supplementary file 2 — Supplementary Dataset [file 41598_2019_46969_MOESM2_ESM.zip › S6 Monte Carlo input & results/EM02_mod_Rev3_20_full_images/IMG0136_401433593.PNG]

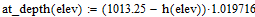

Supplement: Supplementary file 2 — Supplementary Dataset [file 41598_2019_46969_MOESM2_ESM.zip › S6 Monte Carlo input & results/EM02_mod_Rev3_20_full_images/IMG0137_401433593.PNG]
